# Ca^2+^ binding to the C_2_E domain of otoferlin is required for hair cell exocytosis and hearing

**DOI:** 10.1093/procel/pwad058

**Published:** 2023-12-08

**Authors:** Han Chen, Mehar Monga, Qinghua Fang, Loujin Slitin, Jakob Neef, Shashank S Chepurwar, Regina Célia Mingroni Netto, Karina Lezirovitz, Alfredo Tabith, Fritz Benseler, Nils Brose, Kathrin Kusch, Carolin Wichmann, Nicola Strenzke, Barbara Vona, Julia Preobraschenski, Tobias Moser

**Affiliations:** Institute for Auditory Neuroscience and InnerEarLab, University Medical Center Göttingen, 37075 Göttingen, Germany; Collaborative Research Center 889, University of Göttingen, 37075 Göttingen, Germany; Auditory Neuroscience and Synaptic Nanophysiology Group, Max Planck Institute for Multidisciplinary Sciences, 37075 Göttingen, Germany; Göttingen Graduate Center for Neurosciences, Biophysics and Molecular Biosciences, University of Göttingen, 37075 Göttingen, Germany; Collaborative Research Center 889, University of Göttingen, 37075 Göttingen, Germany; Göttingen Graduate Center for Neurosciences, Biophysics and Molecular Biosciences, University of Göttingen, 37075 Göttingen, Germany; Biochemistry of Membrane Dynamics Group, Institute for Auditory Neuroscience, University Medical Center Göttingen, 37075 Göttingen, Germany; Institute for Auditory Neuroscience and InnerEarLab, University Medical Center Göttingen, 37075 Göttingen, Germany; Collaborative Research Center 889, University of Göttingen, 37075 Göttingen, Germany; Auditory Neuroscience and Synaptic Nanophysiology Group, Max Planck Institute for Multidisciplinary Sciences, 37075 Göttingen, Germany; Institute for Auditory Neuroscience and InnerEarLab, University Medical Center Göttingen, 37075 Göttingen, Germany; Göttingen Graduate Center for Neurosciences, Biophysics and Molecular Biosciences, University of Göttingen, 37075 Göttingen, Germany; Molecular Architecture of Synapses Group, Institute for Auditory Neuroscience and InnerEarLab, University Medical Center Göttingen, 37075 Göttingen, Germany; Center for Biostructural Imaging of Neurodegeneration, University Medical Center Göttingen, 37075 Göttingen, Germany; Institute for Auditory Neuroscience and InnerEarLab, University Medical Center Göttingen, 37075 Göttingen, Germany; Collaborative Research Center 889, University of Göttingen, 37075 Göttingen, Germany; Auditory Neuroscience and Synaptic Nanophysiology Group, Max Planck Institute for Multidisciplinary Sciences, 37075 Göttingen, Germany; Collaborative Research Center 889, University of Göttingen, 37075 Göttingen, Germany; Göttingen Graduate Center for Neurosciences, Biophysics and Molecular Biosciences, University of Göttingen, 37075 Göttingen, Germany; Auditory Systems Physiology Group, Institute for Auditory Neuroscience and InnerEarLab, University Medical Center Göttingen, 37075 Göttingen, Germany; Departamento de Genética e Biologia Evolutiva, Centro de Pesquisas sobre o Genoma Humano e Células-Tronco, Instituto de Biociências, Universidade de São Paulo, São Paulo 05508-220, Brazil; Laboratório de Otorrinolaringologia/LIM32, Faculdade de Medicina, Hospital das Clínicas, Universidade de São Paulo, São Paulo, SP 05508-220, Brazil; DERDIC, Pontifícia Universidade Católica de São Paulo, São Paulo 05508-220, Brazil; Department of Molecular Neurobiology, Max Planck Institute for Multidisciplinary Sciences, 37075 Göttingen, Germany; Collaborative Research Center 889, University of Göttingen, 37075 Göttingen, Germany; Department of Molecular Neurobiology, Max Planck Institute for Multidisciplinary Sciences, 37075 Göttingen, Germany; Multiscale Bioimaging Cluster of Excellence (MBExC), University of Göttingen, 37075 Göttingen, Germany; Institute for Auditory Neuroscience and InnerEarLab, University Medical Center Göttingen, 37075 Göttingen, Germany; Functional Auditory Genomics Group, Auditory Neuroscience and Optogenetics Laboratory, German Primate Center, 37077 Göttingen, Germany; Collaborative Research Center 889, University of Göttingen, 37075 Göttingen, Germany; Molecular Architecture of Synapses Group, Institute for Auditory Neuroscience and InnerEarLab, University Medical Center Göttingen, 37075 Göttingen, Germany; Center for Biostructural Imaging of Neurodegeneration, University Medical Center Göttingen, 37075 Göttingen, Germany; Multiscale Bioimaging Cluster of Excellence (MBExC), University of Göttingen, 37075 Göttingen, Germany; Institute for Auditory Neuroscience and InnerEarLab, University Medical Center Göttingen, 37075 Göttingen, Germany; Collaborative Research Center 889, University of Göttingen, 37075 Göttingen, Germany; Auditory Systems Physiology Group, Institute for Auditory Neuroscience and InnerEarLab, University Medical Center Göttingen, 37075 Göttingen, Germany; Institute for Auditory Neuroscience and InnerEarLab, University Medical Center Göttingen, 37075 Göttingen, Germany; Hearing Genomics Group, Institute for Auditory Neuroscience and InnerEarLab, University Medical Center Göttingen, 37075 Göttingen, Germany; Institute of Human Genetics, University Medical Center Göttingen, 37075 Göttingen, Germany; Collaborative Research Center 889, University of Göttingen, 37075 Göttingen, Germany; Biochemistry of Membrane Dynamics Group, Institute for Auditory Neuroscience, University Medical Center Göttingen, 37075 Göttingen, Germany; Multiscale Bioimaging Cluster of Excellence (MBExC), University of Göttingen, 37075 Göttingen, Germany; Institute for Auditory Neuroscience and InnerEarLab, University Medical Center Göttingen, 37075 Göttingen, Germany; Collaborative Research Center 889, University of Göttingen, 37075 Göttingen, Germany; Auditory Neuroscience and Synaptic Nanophysiology Group, Max Planck Institute for Multidisciplinary Sciences, 37075 Göttingen, Germany; Multiscale Bioimaging Cluster of Excellence (MBExC), University of Göttingen, 37075 Göttingen, Germany

## Dear Editor,

**Figure 1.**
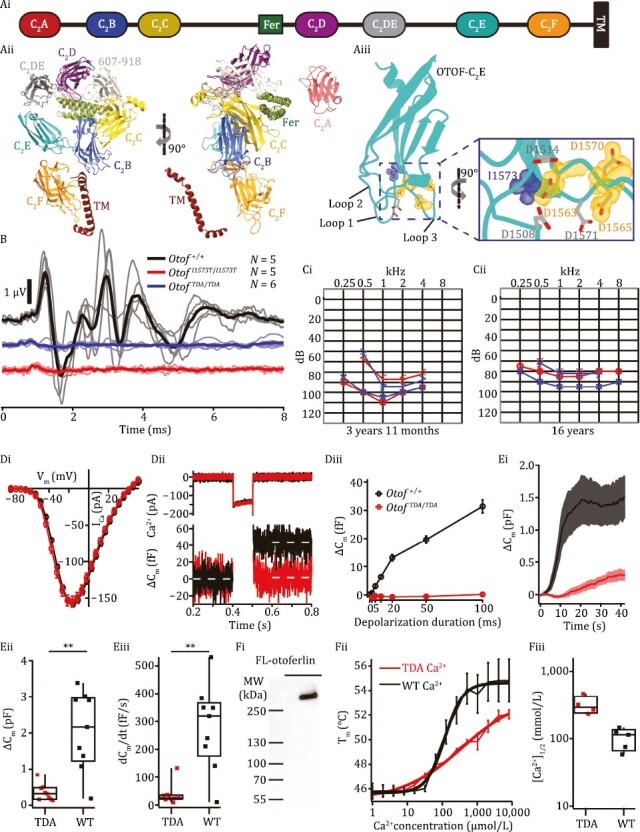
Ca^2+^ binding to the C_2_E domain of otoferlin is required for hair cell exocytosis and hearing. (Ai) Domain organization of otoferlin. Otoferlin is a single-pass transmembrane protein (1,997 amino acids in human) consisting of seven C_2_ domains (C_2_A-F and C_2_DE), one C_2_ domain-like domain (amino acids 607–918, not displayed for simplicity), one FerB domain and a C-terminal transmembrane (TM) domain. (Aii) AlphaFold2-predicted overall structure of the otoferlin protein as a ribbon diagram. (Aiii) Predicted model of otoferlin C_2_E domain: the putative Ca^2+^ binding pocket is formed by top loops 1 and 3. The aspartates substituted by alanine in *Otof*^*TDA*/*TDA*^ and isoleucine 1573 substituted by threonine in *Otof*^*I1573T*/*I1573T*^ are highlighted in orange and blue, respectively. (B) Auditory brainstem responses (ABRs) could not be elicited by 100-dB click stimuli in 9–11-week-old homozygous *Otof*^*TDA*/*TDA*^ mice (middle, individuals: light blue, grand mean: blue) or 5–7-week-old homozygous *Otof*^*I1573T*/*I1573T*^ mice (bottom, individuals: pink, grand mean: red), whereas normal responses were seen in control mice (top, individuals: grey, grand mean: black). (C) Audiograms of the patient at 3 years 11 months (Ci) and 16 years (Cii): red arrow heads and circles represent bone and air conduction thresholds of the right ear, blue arrow heads and crosses represent bone and air conduction thresholds of the left ear. (Di) No difference in the amplitude and voltage-dependence of Ca^2+^ currents between *Otof*^*TDA*/*TDA*^ and *Otof*^+/+^ IHCs (mean ± SEM, *N* = 8 *Otof*^*TDA*/*TDA*^ mice, *n* = 15 *Otof*^*TDA*/*TDA*^ IHCs, *N* = 10 *Otof*^+/+^ mice, *n* = 25 *Otof*^+/+^ IHCs). (Dii) Representative Ca^2+^ currents (top panel) and changes in membrane capacitance (ΔC_m_) (lower panel) of *Otof*^*TDA*/*TDA*^ (red) and control *Otof*^+/+^ IHCs (black) in response to 100 ms depolarization to the voltage where maximum Ca^2+^ currents were elicited (−14 mV). Lack of exocytic ΔC_m_ in *Otof*^*TDA*/*TDA*^ IHCs. (Diii) Lack of exocytic ΔC_m_ as function of duration of depolarization to −14 mV in *Otof*^+/+^ and *Otof*^*TDA*/*TDA*^ IHCs (mean ± SEM). (E) Membrane capacitance recordings with a high concentration of Ca^2+^ (10 mM or mmol/L) in the pipette revealed exocytic ΔC_m_ in both *Otof*^+/+^ and *Otof*^*TDA*/*TDA*^ IHCs (mean ± SEM, *n* = 9 IHCs for both genotypes) (Ei), but with reduced amplitude (Eii) and much slower kinetics (Eiii) in *Otof*^*TDA*/*TDA*^ IHCs. (Fi) Western lot showing affinity-purified full-length (FL) TDA-otoferlin from insect cells. MW: molecular weight. (Fii) Ca^2+^ binding induced thermal stabilization of WT-otoferlin requires a lower Ca^2+^ concentration than for TDA-otoferlin, suggesting a reduction of the Ca^2+^-binding affinity in TDA-otoferlin (Fiii).

**Figure 2.**
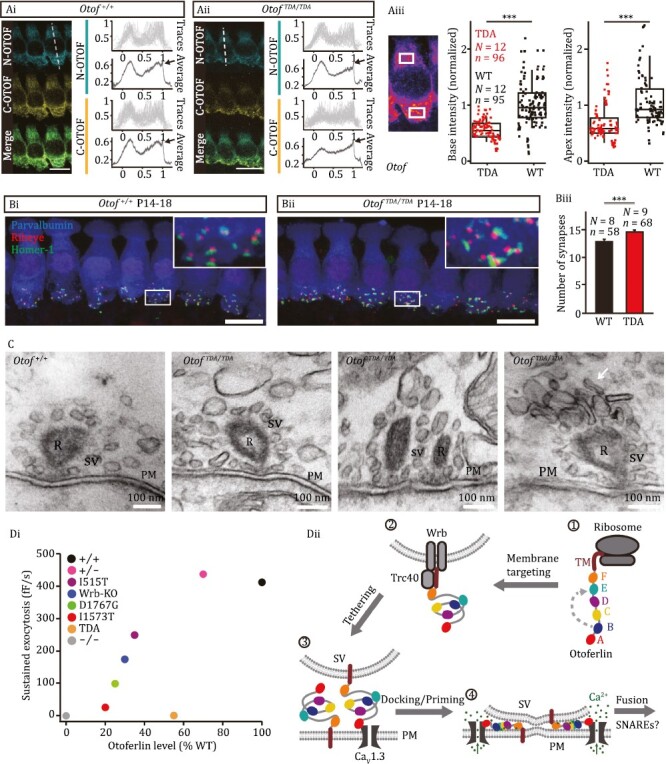
Reduced otoferlin protein levels upon C_2_E mutagenesis but maintained ribbon synapses. (Ai) Staining with both N-terminal and C-terminal antibodies showed the expected broad otoferlin distribution in IHCs except for the nucleus. Line profile analysis (from apex to base, indicated as a white dashed line) revealed an enrichment of otoferlin near the plasma membrane for IHCs of both *Otof*^*TDA*/*TDA*^ (Aii) and littermate control (Ai) mice. Scale bar, 10 μm. (Aiii) Otoferlin immunofluorescence intensity averaged over basal and apical intracellular regions of interest (white boxes) indicated reduced otoferlin levels in *Otof*^*TDA*/*TDA*^ IHCs compared to littermate control IHCs (***P < 0.001, Wilcoxon Rank test). Box and whisker plots represent median, 25th and 75th, as well as 10th and 90th percentiles. (B) Maximum-intensity projections of confocal stacks of postnatal day 14–18 (P14–18) *Otof*^+/+^ (Bi) and *Otof*^*TDA*/*TDA*^ IHCs (Bii) following immunolabeling for parvalbumin (blue), ribeye (red), and homer-1 (green). Juxtaposed ribeye and homer-1 immunofluorescent spots indicate ribbon synapses. Scale bar, 10 μm. (Biii) The number of ribbon synapses per IHC was slightly higher for *Otof*^*TDA*/*TDA*^ mice, *** *P* < 0.001. (C) Representative electron micrographs from the different *Otof*^*TDA*/*TDA*^ and *Otof*^+/+^ ribbon synapses. R: ribbon, SV: synaptic vesicle, PM: plasma membrane. (Di) Data from (Pangrsic et al, 2010; Strenzke et al, 2016; Vogl et al, 2016) and this study. Data obtained on *Otof*^*D1767G*/*D1767G*^ and *Otof*^*I515T*/*I515T*^ IHCs initiated the notion that SV replenishment has a more stringent requirement of otoferlin than fusion. *Otof*^*TDA*/*TDA*^ and *Otof*^*I1573T*/*I1573T*^ IHCs lack exocytosis as much as *Otof*^−/−^ despite residual otoferlin expression (55% and 20%, respectively). (Dii) Postulated roles of the C_2_F domain in membrane targeting (modified from Vogl et al. (2016)) and of the C_2_E domain in Ca^2+^ dependent SV priming and Ca^2+^ triggered SV fusion (C_2_E) in a speculative model of otoferlin function at IHC synapses. (1) Otoferlin is translated, the C_2_E domain is important for the folding of otoferlin via intramolecular interaction with the C_2_B domain. (2) The transmembrane recognition complex (TRC40) pathway mediates the membrane insertion of otoferlin. (3) Otoferlin contributes to SV tethering to the AZ membrane, potentially involving homophilic and heterophilic protein–protein interaction and binding to the target membrane (SV or AZ). (4) Upon Ca^2+^ rise during depolarization, the ring of C_2_ domains inserts into the target membrane via top-loop Ca^2+^–phospholipid interaction. This could further draw together SV and AZ membrane into a docked and potentially primed state. Alternatively, upon a Ca^2+^ rise, the C_2_E domain might disengage from C_2_B and become available for Ca^2+^–phospholipid interaction, thereby initiating the insertion of the C_2_ domains into the target membrane. This, as well as the precise molecular mechanism of the subsequent membrane fusion, remains to be elucidated.

Afferent synapses of cochlear inner hair cells (IHCs) employ a unique molecular machinery (see extended background in Supplementary Materials). Otoferlin is a key player in this machinery and its defects cause human auditory synaptopathy (Moser and Starr, 2016). Otoferlin, a tail-anchored (Vogl et al., 2016) multi-C_2_-domain protein (Fig. 1Ai) specific to hair cells (Roux et al., 2006), is a member of the ferlin protein family involved in membrane trafficking and repair that are of major disease relevance (Pangršič et al., 2012), also see Supplementary Materials. Otoferlin is distributed broadly within IHCs (Fig. 2Ai-Aiii; Pangrsic et al., 2010; Roux et al., 2006). Otoferlin seems to have a multifaceted role in the synaptic vesicle (SV) cycle at IHC active zones (AZs) (Moser and and Starr, 2016), serving as (i) candidate Ca^2+^ sensor of SV fusion (Johnson and Chapman, 2010; Michalski et al., 2017; Roux et al., 2006) and (ii) promoter of Ca^2+^ dependent SV replenishment at the release sites (Pangrsic et al., 2010;Strenzke et al., 2016; Vogl et al., 2016), and mediating exocytosis–endocytosis coupling (Jung et al., 2015). AlphaFold2 prediction of otoferlin structure (Figs. 1Aii and S1A) suggests that the Ca^2+^ binding top loops of the C_2_E domain contribute to the ring-like tertiary structure by intramolecular interaction with the C_2_B domain. This likely involves electrostatic interactions and can potentially be modulated by Ca^2+^ bound to C_2_E. Ca^2+^ binding by C_2_E likely involves the highly conserved aspartates D1508, D1514, D1563, D1565, and D1570/D1571 (Figs. 1Aiii and S1B). Reported [Ca^2+^]_1/2_ values of C_2_E amount to 25 µmol/L and 7.5 µmol/L in the absence and presence of phospholipids, respectively (Johnson and Chapman, 2010). Here, we targeted three of them for alanine substitution by CRISPR/Cas9 genome-editing and generated homozygous *Otof*^*D1563*/*1565*/*1570A*^ mice (which we abbreviate *Otof*^*TDA*/*TDA*^, for “triple D (aspartate) to A (alanine)”). In addition, we introduced a human mutation (I1573T, *Otof*^*I1573T*/*I1573T*^ mice) in the immediate proximity of the Ca^2+^ binding top loop aspartates (Fig. 1Aiii) that we expect to affect Ca^2+^/phospholipid binding. Correct editing was confirmed by amplifying genomic fragments by location-specific PCR and Sanger sequencing the corresponding PCR amplicons. Recordings of ABRs indicated a loss of synchronized activation of spiral ganglion neurons (SGNs) (wave I reflecting the SGN compound action potential (CAP)) and propagated neural activity along the early auditory pathway despite sizable summating potential (primarily reflecting the IHC receptor potential) in homozygous *Otof*^*TDA*/*TDA*^ and *Otof*^*I1573T*/*I1573T*^ mice (Fig. 1B). Distortion product otoacoustic emissions (DPOAEs) were observed in both mutants indicating intact cochlear amplification by outer hair cells (OHCs, Fig. S2). Lack of ABR despite intact OHC function signifies auditory synaptopathy or neuropathy (Moser and Starr, 2016).

Lack of ABR was also reported for a nine-year-old child homozygous for *OTOF*^*I1573T*^ (Yildirim-Baylan et al., 2014). Here, we report the clinical data of a Brazilian individual with a homozygous *OTOF*^*I1573T*^ variant (Fig. S3A and further Suppl.). Following an initial suspicion of hearing impairment around 12 months of age by the mother, the child was later clinically diagnosed at the age of 3 years 11 months and had delayed speech acquisition. At the age of 4 years, a lack of ABR was identified (tested up to 85dB (HL), not shown), despite otoacoustic measured as membrane capacitance (Fig. S3B). This is consistent with data obtained from the *Otof*^*I1573T*/*I1573T*^ mouse model and supports the notion of an auditory synaptopathy with intact OHC function but impaired synaptic sound encoding. Comparing the first (3 years 11 months, Fig. 1Ci) and last (16 years, Fig. 1Cii) available audiograms, hearing impairment remained stable, at moderate to profound severity, contrasting the previous notion of progressive hearing impairment in homozygous *OTOF*^*I1573T*^ patients (Yildirim-Baylan et al., 2014). The patient currently uses hearing aids, which improve his hearing sensitivity by 35 dB. The patient had normal motor neurodevelopment and no other pathologies were found by routine diagnostics including electroencephalography and magnetic resonance imaging of the brain. At 5 years of age, the patient was referred for genetic testing where sequencing of *OTOF* uncovered the homozygous *OTOF*^*I1573T*^ variant. His mildly hearing impaired parents were each confirmed as heterozygous carriers of the *OTOF*^*I1573T*^ variant. Re-evaluation of the genetic variant in light of the *Otof*^*I1573T*/*I1573T*^ mice definitively reclassifies the variant from likely pathogenic (range: 6–9 points) (PS1_Strong, PM2_Supporting, PM3_Supporting, PP3_Supporting, PP4_Supporting, 8 points) to pathogenic (≥ 10 points) (adding PS3_Strong, 12 points).

We then took advantage of the novel mouse mutants to investigate the precise cellular and synaptic mechanisms of impaired sound encoding upon C_2_E mutation. As reduced or jittered SGN spiking may fail to elicit detectable ABRs, we attempted recordings of sound-evoked neuronal spiking activity *in vivo* in the region where the auditory nerve enters the cochlear nucleus. These recordings assay synaptic sound encoding at single afferent IHC synapses with great precision and have been instrumental in revealing a function of otoferlin in SV replenishment to the readily releasable pool (Pangrsic et al., 2010; Strenzke et al., 2016). However, in contrast to wild-type (WT) mice, recording from *Otof*^*TDA*/*TDA*^ mice did not detect any obvious sound-evoked neural activity in that region. In a total recording duration of 23.5 hours in five *Otof*^*TDA*/*TDA*^ mice, we never encountered any sound-evoked action potentials. For comparison, in a WT mouse dataset, we, on average, recorded one auditory nerve fiber and one other sound-responsive neuron (e.g., bushy cells and multipolar cells of the cochlear nucleus) per 90 min of the running experiment. These data support the notion of a major sound encoding failure at afferent IHC synapses of *Otof*^*TDA*/*TDA*^ mice *in vivo*.

To further address the effects of the C_2_E mutations on presynaptic IHC function, we performed perforated-patch recordings from IHCs of *Otof*^*TDA*/*TDA*^ and *Otof*^*I1573T*/^^*I1573T*^ mice in the third postnatal week (postnatal day 14–18 (P14–18)). Despite normal voltage-gated Ca^2+^ influx (Fig. 1Di and Dii), which is in line with the normal number of afferent synapses (see Fig. 2), both *Otof*^*TDA*/*TDA*^ (Fig. 1D) and *Otof*^*I1573T*/*I1573T*^ IHCs (Fig. S4) lacked exocytosis measured as membrane capacitance (C_m_) increments (Fig. 1Dii and Diii) with residual IHC exocytosis of both mutants being comparable to that of *Otof*^−/−^ IHCs (Pangrsic et al., 2010; Roux et al., 2006). Considering that the otoferlin levels of *Otof*^*TDA*/*TDA*^ and *Otof*^*I1573T*/*I1573T*^ IHCs (55% and 20%, respectively, see Figs. 2 and S8) are comparable to *Otof*^*I515T*/I*515T*^ (Strenzke et al., 2016) and *Otof*^*D1767G*/*D1767G*^ (Pangrsic et al., 2010) IHCs, respectively, which both show intact phasic Ca^2+^ exocytosis, we aimed to address the possibility that impaired Ca^2+^ binding to otoferlin underlies the lack of Ca^2+^-influx-triggered exocytosis in *Otof*^*TDA*/*TDA*^ IHCs. We reasoned that a lowering of Ca^2+^ affinity due to the C_2_E mutations could render the [Ca^2+^] achievable with voltage-gated Ca^2+^ influx at the IHC SV release sites (likely between 50 and 150 µmol/L, see Suppl.) insufficient to serve as a trigger of SV fusion. We expected that uncaging of Ca^2+^-loaded DM-Nitrophen reaching [Ca^2+^] up to 100 µmol/L in IHCs (Beutner et al., 2001) would not help overcome the postulated gap to the [Ca^2+^] required for fusion.

Therefore, we turned to ruptured-patch C_m_ recordings with 10 mmol/L [Ca^2+^] in the pipette, to test for potential exocytosis at higher [Ca^2+^] (Figs. 1Ei–Eiii and S5). In line with the above hypothesis, we could elicit a C_m_ increase in *Otof*^*TDA/TDA*^ IHCs, which, however, proceeded with slower kinetics and reached lower maximal amplitudes than in *Otof*^+/+^ IHCs. *Otof*^+/+^ IHCs typically showed an onset of the exocytic C_m_ rise around 1–5 s after membrane rupture, that peaked at or above 1 pF, followed by a likely endocytic C_m_ decline while the other outputs of the impedance analysis (series and membrane resistance) remained largely constant, confirming the specificity of the C_m_ change. Despite comparable exposure to the pipette [Ca^2+^] given similar series resistance (12.5 ± 1.3 MΩ for *Otof*^*TDA*/*TDA*^ IHCs, *n* = 9 vs. 12.7 ± 1.1 MΩ for *Otof*^+/+^ IHCs, *n* = 9), the C_m_ rise of *Otof*^*TDA*/*TDA*^ IHCs started around 10 s after membrane rupture and stayed below 1 pF for the duration of the recording.

To further scrutinize the functional alterations caused by the *Otof*^*TDA*/*TDA*^ mutation, we performed *in vitro* experiments on purified full-length otoferlin. Full-length otoferlin was obtained at high purity from SF9 insect cells using affinity and ion exchange chromatography (Figs. 1Fi–Fiii and S6). We then subjected TDA-otoferlin to nano differential scanning fluorimetry (nanoDSF) and found Ca^2+^, but not Mg^2+^, to increase the melting temperature (Tm) in a dose-dependent manner (Fig. S6B and S6C). This suggests TDA-otoferlin still and selectively binds Ca^2+^. We then analyzed Ca^2+^ dependence of Tm for TDA-otoferlin in comparison to WT-otoferlin, both in the absence of phospholipids (Fig. 1Fi and Fii). We found that the estimated [Ca^2+^]_1/2_ of TDA-otoferlin was significantly higher than for WT-otoferlin ([Ca^2+^]_1/2_ = 321.6 ± 52.2 µmol/L for TDA-otoferlin vs. [Ca^2+^]_1/2_ = 104.5 ± 16.3 µmol/L for WT-otoferlin, mean ± SEM, *P* < 0.01) which is suggestive of a reduced Ca^2+^ binding affinity in the mutant (Fig. 1Fiii). In summary, analyses of the C_2_E mutations indicates an essential role of the domain for Ca^2+^ triggered exocytosis in IHCs likely involving Ca^2+^ sensing for SV fusion and/or replenishment.

We then turned to semiquantitative analysis of otoferlin immunofluorescence (antibodies to C- and N-terminal epitopes) in the mutant mice which revealed a reduced expression but near normal subcellular distribution of otoferlin in *Otof*^*TDA*/*TDA*^ IHCs (Fig. 2Ai and Aii). Line profiles, drawn from apex to base, showed the typical apical and basal maxima of otoferlin immunofluorescence (with the center dip corresponding to the nucleus). The fluorescence peak at the basal edge (arrows in A_i_ and A_ii_) corresponds to otoferlin expression in the plasma membrane which was maintained in *Otof*^*TDA*/*TDA*^ IHCs. In order to further scrutinize the subcellular abundance of otoferlin, we quantified otoferlin immunofluorescence in apical and basal regions of interest (Fig. 2Aiii), which revealed a reduction to 60.4% ± 4.7% and 55.3% ± 3.2% (*n* = 96 IHCs from *N* = 12 *Otof*^*TDA*/*TDA*^ mice, *n* = 95 IHCs from *N* = 12 *Otof*^+/+^ mice), respectively. Using quantitative PCR of mRNA obtained from *Otof*^*TDA/TDA*^ organs of Corti, we found increased otoferlin mRNA levels suggesting a compensatory upregulation of *Otof* transcription (Fig. S7). *Otof*^*I1573T/I1573T*^ IHCs showed a more drastic reduction of otoferlin levels (Fig. S8A–D) to 25.20% ± 2.28% and 20.54% ± 2.70% in apical and basal regions (*Otof*^*I1573T*/*I1573T*^: *n* = 50 IHCs, *N* = 6 mice; *Otof*^+/+^: *n* = 125 IHCs, *N* = 15 mice) with comparable mRNA levels suggesting reduced protein abundance despite intact *Otof* transcription (Fig. S8E and S8F).

Next, we evaluated the IHC-SGN connectivity using immunohistochemistry for IHC synaptic ribbons and for the postsynaptic density of SGNs. Counting juxtaposed pairs of ribbons and postsynaptic densities (Khimich et al., 2005) indicated that ribbon synapses were present in comparable number in *Otof*^*TDA*/*TDA*^ IHCs (Fig. 2B) and *Otof*^*I1573T*/*I1573T*^ IHCs at 3 and 4 weeks of age (Fig. S9). We then addressed the synaptic ultrastructure of *Otof*^*TDA*/*TDA*^ IHCs using conventional embedding and transmission electron microscopy of ultrathin sections (Fig. 2C). In keeping with immunofluorescence microscopy, we found AZs with anchored ribbons and comparable ribbon area in *Otof*^*TDA*/*TDA*^ IHCs (Fig. S10A). Counting SVs revealed normal total SV counts per ribbon and a slightly lower SV density at AZs of *Otof*^*TDA*/*TDA*^ IHCs (Fig. S10B and S10C, *P* < 0.01). We then focused on the two morphological SV pools, ribbon-associated (RA)-SVs and membrane-proximal (MP)-SVs, as was done previously (Jean et al., 2018). The MP- and RA-SVs of *Otof*^*TDA*/*TDA*^ IHCs were unaltered in numbers (Fig. S10D and S10E). Finally, we observed pleomorphic vesicles at *Otof*^*TDA*/*TDA*^ ribbon synapses (Fig. 2C, white arrow). These vesicles were found at the ribbon as well as at the AZ membrane and might represent endosome-like vacuoles (Jung et al., 2015).

In summary, detailed molecular and cellular analysis of the novel mouse mutants aimed to disrupt Ca^2+^ binding of the C_2_E domain revealed a lack of Ca^2+^ influx-triggered IHC exocytosis despite considerable expression of otoferlin remaining in IHCs. The levels of otoferlin in the basolateral IHC pole were reduced from moderately in *Otof*^*TDA*/*TDA*^ to profoundly in *Otof*^*I1573T*/ *I1573T*^. Different from other *Otof* mouse mutants, we found the afferent IHC synapses to be maintained in the two novel mutants. Moreover, the ultrastructure of the AZs of *Otof*^*TDA*/*TDA*^ IHCs appeared largely intact. In keeping with the hypothesis that otoferlin acts as a Ca^2+^ sensor in IHC exocytosis, we found Ca^2+^ binding by TDA-otoferlin to be altered and exposure to millimolar [Ca^2+^] via the patch-pipette to elicit exocytosis in IHCs, albeit with a drastically reduced rate. We conclude that the C_2_E domain is critical for otoferlin function. Aside from its likely role in Ca^2+^ triggered membrane fusion, the Ca^2+^-bound pocket of the C_2_E domain might be important for the tertiary structure of otoferlin, e.g., by interacting with the N-terminal C_2_B domain.

Thus far, *in vitro* approaches to structure-function analysis of full-length otoferlin have been largely lacking, to our knowledge, partly due to the difficulty in purifying full-length otoferlin at good quantity and quality. Therefore, modeling human *OTOF* missense mutations in mice and testing hypotheses regarding otoferlin function using site-directed mutagenesis and multiscale analyses of the auditory system have been essential to advance our understanding of the role of otoferlin in the physiology and pathophysiology of afferent synapses between IHCs and SGNs. Compared to published data on mutations affecting the C_2_C domain (Michalski et al., 2017; Strenzke et al., 2016) where ABRs are preserved albeit with reduced amplitude, ABRs were more strongly affected upon manipulation of the most C-terminal stretch of otoferlin including the C_2_E and C_2_F domains (Pangrsic et al., 2010; Vogl et al., 2016). The lack of ABRs in homozygous *OTOF*^*I1573T*^ patients and in *Otof*^*I1573T*/ *I1573T*^ mice contrast the psychophysical evidence of residual acoustic sensitivity in the human patients. Pure tone audiometry indicated a progressive (mild to severe) hearing impairment in the four pediatric cases of Turkish descent reported by Yildirim-Baylan et al. (2014), while a profound hearing impairment was found in a Japanese toddler (Iwasa et al., 2022), and moderate to profound hearing impairment in the patient described in this study. This discrepancy is consistent with the notion that recordings of ABR and SGN compound action potential more sensitively report impaired synchronous transmission at afferent synapses between IHCs and SGNs than pure tone audiograms (Moser and Starr, 2016).

However, speech understanding is strongly impaired and typically not improved by hearing aids (currently used by the homozygous *OTOF*^*I1573T*^ patient reported here), which has resulted in fitting of cochlear implants (Moser and Starr, 2016). Regarding the apparent discrepancy between the near complete lack of exocytosis in *Otof*^*I1573T*/*I1573T*^ IHCs and the residual hearing in the homozygous*OTOF*^*I1573T*^ patients, we speculate that our patch-clamp analysis of exocytosis and the recordings of ABR might not be ideally suited to study the residual synaptic transmission at the afferent synapses of *Otof*^*I1573T*/*I1573T*^ IHCs. Indeed, the residual synaptic transmission of the afferent IHC synapse, despite the lack of ABR, was indicated by recordings of exitatory postsynaptic currents and sound-evoked SGN firing in *Otof* mutant mice (Pangrsic et al., 2010), and we might have missed such residual firing in our attempted SGN recordings from *Otof*^*TDA*/*TDA*^ mice. Further evidence for partial otoferlin functionality in the two alleles targeting C_2_E studied here is the maintenance of IHC synapses, which are partially lost in *Otof*^−/−^ [by ~50% (Roux et al., 2006), also see Supplementary Materials], and *Otof*^*D1767G*/*D1767G*^ mice [by ~20%, (Pangrsic et al., 2010)].

Candidate molecular disease mechanisms causing hearing impairment in otoferlin-related auditory synaptopathies include alterations of translation, membrane and subcellular targeting, Ca^2+^-, lipid-, and protein-binding, and stability or turnover of otoferlin. Our overall conclusion from analyzing the novel mouse mutants and the purified TDA-otoferlin is that C_2_E domain contributes to Ca^2+^ sensing for SV fusion. However, the mutations caused additional alterations such as reduced otoferlin levels and/or disturbed subcellular otoferlin distribution which need to be considered carefully. Previous analysis of mouse mutants with reduced levels of otoferlin, be it due to *Otof* mutation (Jung et al., 2015; Pangrsic et al., 2010; Strenzke et al., 2016) or disruption of interacting proteins (Jung et al., 2015; Vogl et al., 2016), showed that Ca^2+^ triggered SV fusion is robust down to at least 25% of WT levels (*Otof*^*D1767G*/*D1767G*^) while the rate of SV replenishment is more sensitive to reduced otoferlin levels (Fig. 2Di). Otoferlin levels in *Otof*^*TDA*/*TDA*^ IHCs amounted to ~55% of WT levels. This exceeds otoferlin levels in *Otof*^*D1767G*/*D1767G*^ and *Otof*^*I515T*/*I515T*^ IHCs that showed intact SV fusion. Hence, the near complete lack of Ca^2+^-influx-triggered SV exocytosis of *Otof*^*TDA*/*TDA*^ IHCs cannot be merely due to the reduced otoferlin levels. Instead, it likely reflects an impairment of Ca^2+^-sensing for SV fusion, although other possibilities cannot be ruled out unequivocally.

Support for the notion that Ca^2+^ binding to the C_2_E domain participates in Ca^2+^ sensing for SV fusion in IHCs includes the reduction of the apparent Ca^2+^ affinity of TDA-otoferlin as indicated by nano-DSF. These experiments did not reveal a significant reduction of thermal stability, which argues against a major alteration of the tertiary structure of TDA-otoferlin. We propose that the reduced Ca^2+^ affinity of TDA-otoferlin places the Ca^2+^ dependence of exocytosis in *Otof*^*TDA*/*TDA*^ IHCs outside the range of [Ca^2+^] achieved by voltage-gated Ca^2+^ influx and Ca^2+^ uncaging [up to ~100 μmol/L Beutner et al. (2001)]. In a first attempt to address this issue, we used dialysis of high [Ca^2+^] from the patch-pipette, which indeed unlocked some exocytosis in *Otof*^*TDA*/*TDA*^ IHCs albeit with slower kinetics and lower amplitude as compared to *Otof*^+/+^ IHCs. These results are consistent with the notion that Ca^2+^ binding to the C_2_E domain contributes to Ca^2+^ sensing for SV fusion in IHCs. However, they do not provide definitive proof. Ideally, the expected changes in the Ca^2+^ dependence of SV fusion should be tested for by measurements of initial release from the readily releasable pool of SVs for step-like elevations of [Ca^2+^] at the release sites to different levels. Future experiments should involve more subtle alterations of the C_2_E-top loop function by substituting only one or two of the putative Ca^2+^ coordinating aspartate residues. This might place the Ca^2+^ dependence of IHC exocytosis back into the range of time-resolved [Ca^2+^] changes achievable by voltage-gated Ca^2+^ influx or Ca^2+^ uncaging.

We summarized our interpretation of the current findings in a speculative model (Fig. 2D) that highlights the role of the C-terminal C_2_E domain. The presence of otoferlin in the SV and presynaptic plasma membranes has been demonstrated by immunofluorescence and immunogold-labeling (Pangrsic et al., 2010; Roux et al., 2006; Strenzke et al., 2016). Otoferlin contributes to SV tethering to the AZ membrane (see Supplementary Material) potentially involving homophilic and heterophilic protein-protein interaction and binding to the target membrane (SV or AZ), which we consider a loose and reversible state of SV immobilization. Ca^2+^ binding to C_2_E and other C_2_ domains might then draw the SV and AZ membranes further together, e.g., by forming a ring-like tertiary core structure that inserts into the target membrane via C_2_ domain-Ca^2+^-phospholipid interaction (Fig. 1) and by “kinking” the membrane-bound ring relative to the transmembrane domain. This might correspond to the morphologically docked SV state, which is rare and likely transient at resting IHC AZs (Chakrabarti et al., 2022) and thus prepare fusion by generating curvature in both the SV and AZ membranes. We speculate that SVs proceed from there to fusion, if provided with sufficient [Ca^2+^], or undock again. Future work will be required to test this model and to reveal whether the actual SV fusion is then mediated by SNARE-based machinery or executed by an unconventional ferlin-based fusion machinery.

## Supplementary information

The online version contains supplementary material available at https://doi.org/10.1093/procel/pwad058.

pwad058_suppl_Supplementary_Material

## Footnotes

We are grateful for the family’s participation in our study. We thank Dayane Bernardino Cruz for technical assistance; Dr. Beatriz C.A. Mendes and professionals of DERDIC for the clinical studies. We thank Dr. Thomas Weber for his contribution in the initial phase of the project, and Dr. Anna Vavakou for her contributions to ABR and single auditory nerve fiber recordings. We thank Nadine Dietrich, Sandra Gerke, Daniela Gerke, Olivia Lange, Sina Langer, Christiane Senger-Freitag and Christiane Harenberg for expert technical assistance and the transgenic mouse team for excellent service.

This work was supported by the Deutsche Forschungsgemeinschaft (DFG, German Research Foundation), under Germany’s Excellence Strategy — EXC 2067/1- 390729940 (to T.M., N.B. and J.P.), via the Collaborative Research Center 889 (to N.S., C.W., J.P., N.B. and T.M.), via DFG VO 2138/7 ‐ 1 (to B.V.), and via the Leibniz Program (to T.M.). In addition, this research was supported by Fondation Pour l’Audition (FPA RD-2020-10) to T.M. and Fundação de Amparo à Pesquisa do Estado de São Paulo (CEPID 2013/08028-1) to R.C.M.N..

The authors declare that they have no conflict of interest. This study was approved by the Institutional Ethics Committee of the Biosciences Institute at the University of S ão Paulo. Written informed parental consent was obtained. The affected child underwent ABR, otoacoustic emission and cochlear microphonic recordings, age-appropriate audiometry, brain MRI and electroencephalography.

The c. 4718T>C (p.Ile1573Thr) variant has been deposited in ClinVar with the accession SCV001762966.1.

T.M. and H.C. designed the study with contributions of other authors. H.C. performed mRNA analysis, protein analysis, immunohistochemistry, confocal microscopy, hair cell physiology and data analysis (with contribution from J.N.). Q.F. hair cell physiology and data analysis. T.M. supervised H.C. and Q.F. with contribution of K.K. (mRNA analysis). M.M., supervised by J.P., performed protein purification and analysis together with H.C.. L.S. performed electron microscopy under supervision of C.W., F.B. and N.B. performed mouse mutagenesis (with the transgenic mouse team) and genotyping. R.C.M.N., K.L., A.T., and B.V. performed the human genetics work. S.S.C. performed systems physiology data acquisition and analysis under supervision of N.S. T.M. and H.C. prepared the manuscript with contributions from all other authors.
