# Supplementary material for: Ca2+ binding to the C2E domain of otoferlin is required for hair cell exocytosis and hearing
Source: Protein Cell. 2023 Dec 8;15(4):305–12. doi: 10.1093/procel/pwad058 (PMC10984619; doi:10.1093/procel/pwad058)
Supplement: pwad058_suppl_Supplementary_Material [file pwad058_suppl_supplementary_material.pdf]

Supplementary Material

List of supplementary material:

1. Extended Background
2. Materials and Methods
3. Figure S1. Predicted structure of otoferlin and amino acids of C_2E targeted by mutagenesis. Related to Figure 1.
4. Figure S2. Mutation of the C_2E domain causes synaptopathic hearing impairment in mice. Related to Figure 1.
5. Figure S3. A human mutation of the C_2E domain causes synaptopathic hearing impairment. Related to Figure 1**.**
6. Figure S4. Ca^{2+} influx triggered exocytosis is largely abolished in *Otof*$^{I1573T/I1573T}$ IHCs. Related to Figure 1.
7. Figure S5. Partial restoration of exocytosis in *Otof*$^{TDA/TDA}$ IHCs upon Ca^{2+} dialysis. Related to Figure 1.
8. Figure S6. TDA-otoferlin has a lower apparent affinity to Ca^{2+}. Related to Figure 1.
9. Figure S7. Reduction of otoferlin protein in *Otof*$^{TDA/TDA}$ IHC levels despite normal mRNA levels in *Otof*$^{TDA/TDA}$. Related to Figure 2
10. Figure S8. Reduction of otoferlin protein levels despite normal mRNA levels in *Otof*$^{I1573T/I1573T}$ IHCs. Related to Figure 2.
11. Figure S9. The number of ribbon synapses in *Otof*$^{TDA/TDA}$ and *Otof*$^{I1573T/I1573T}$ IHCs remain unchanged. Related to Figure 2.
12. Figure S10. Largely unaltered ultrastructure of ribbon synapses in *Otof*$^{TDA/TDA}$ IHCs. Related to Figure 2.
13. Extended Discussion

Extended Background

Deciphering the molecular physiology of sound encoding at the afferent IHC synapses is an ongoing effort (Johnson *et al*, 2019; Moser, 2020; Rutherford *et al*, 2021; Safieddine *et al*, 2012). For example, multidomain active zone (AZ) scaffold proteins bassoon (Khimich *et al*, 2005; Buran *et al*, 2010), piccolino (Müller *et al*, 2019), RIM (Rab-3-interacting molecule)2 and RIM3 (Jung *et al*, 2015a; Picher *et al*, 2017b), RIM-BP2 (Krinner *et al*, 2017, 2021), and the ribbon specific protein RIBEYE (Jean *et al*, 2018; Becker *et al*, 2018) were shown to be required for normal sound encoding. Surprisingly, the presence and function of the SNARE (soluble N-ethylmaleimide-sensitive-factor attachment receptor)-based neuronal synaptic vesicle (SV) fusion machinery (Nouvian *et al*, 2011; Calvet *et al*, 2022) and its regulators, such as complexins (Strenzke *et al*, 2009), synaptotagmins 1 and 2 (Beurg *et al*, 2010; Reisinger *et al*, 2011; Johnson *et al*, 2010), or Munc13/CAPS (Ca^{2+}-dependent activator proteins for secretion) priming factors (Vogl *et al*, 2015) at IHC synapses is less clear. This suggests a deviation of IHC AZs from the molecular machinery of conventional synapses. In line with this notion, human genetics studies have discovered mutations in a number of deafness-associated genes that cause auditory synaptopathy often without further neurological deficits.

Such genetic auditory synaptopathies (Moser *et al*, 2006; Santarelli *et al*, 2015; Moser & Starr, 2016) can involve deficiency or dysfunction of the $Ca_V1.3$ Ca^{2+} channel (Baig *et al*, 2011) or its modulators CaBP2 (Schrauwen *et al*, 2012; Picher *et al*, 2017a), harmonin (Gregory *et al*, 2011), and GIPC3 (Ohn *et al*, 2016), as well as the vesicular glutamate transporter 3 (Greene *et al*, 2001), and, notably, otoferlin (Yasunaga *et al*, 1999; Roux *et al*, 2006). Otoferlin, is a member of the ferlin protein family also including dysferlin and myoferlin that, as otoferlin, are of major disease relevance (Lek *et al*, 2012; McNeil & Kirchhausen, 2005; Pangršič *et al*, 2012). Otoferlin seems to have a multifaceted role in IHC transmitter release (Moser & Starr, 2016) but dissecting the function of otoferlin has been hampered by the lack of model systems beyond zebrafish and mouse hair cells and difficulties in purifying full-length otoferlin at sufficient quantity and quality. As a result, many *in vitro* studies employed fragments with individual or subsets of C_2 domains (Roux *et al*, 2006; Johnson & Chapman, 2010; Pangrsic *et al*, 2010; Helfmann *et al*, 2011; Jung *et al*, 2015b; Michalski *et al*, 2017; Golbek *et al*, 2019; Hams *et al*, 2017). Moreover, so far only very few human missense mutations have been studied in detail (Strenzke et., 2016: I515T, C_2C domain; Vogl et al., 2016, I1967del, linker to transmembrane domain).

Materials and Methods

Patient recruitment, clinical evaluation, and molecular genetic testing

This study was approved by the Institutional Ethics Committee of the Biosciences Institute at the University of São Paulo. Written informed parental consent was obtained. The affected child underwent ABR, otoacoustic emission and cochlear microphonic recordings, age-appropriate audiometry, brain MRI and electroencephalography. Sanger sequencing of the complete coding *OTOF* sequence was performed as previously described (Romanos *et al*, 2009). Following parental testing, the variant was re-evaluated using the American College of Medical Genetics and Genomics guidelines for hereditary hearing loss (Oza *et al*, 2018) with use of the public version of VarSome (Kopanos *et al*, 2019).

Animals

Two knock-in mouse lines, $Otof^{D1563/1565/1570A}$ (i.e. $Otof^{TDA}$) and $Otof^{I1573T}$were generated by site-directed CRISPR-Cas9 mutagenesis. Mice of either sex, aged between 2 weeks and 11 months, were used for experiments. Mice were kept in individually ventilated cages with environmental enrichment (carboard rolls, wood wool) on a 12/12 h day/night cycle in the animal facility of the Max Planck Institute for Multidisciplinary Sciences. All experiments complied with national animal care guidelines and were approved by the University of Göttingen Board for Animal Welfare and the Animal Welfare Office of the State of Lower Saxony (AZ 19/3133 and AZ 19/3134).

Structure prediction

Otoferlin structure was predicted by AlphaFold 2 (Jumper *et al*, 2021), which can be directly downloaded in UniProt (Q9HC10, uniprot.org). The protein structure figures were prepared by using the program PyMOL or ChimeraX.

Generating *Otof* mouse mutants using CRISPR/Cas9 gene editing

Superovulated C57BL/6N females were mated with C57BL/6N males and fertilized eggs collected. In-house prepared CRISPR reagents (hCAS9_mRNA, sgRNAs, and either dsDNA or long oligonucleotides were used as repair templates containing the desired mutation), or preformed Cas9_sgRNA RNP complexes were microinjected into the pronucleus and the cytoplasm of zygotes at the pronuclear stage using an Eppendorf Femtojet. Note that all nucleotide based CRISPR-Cas9 reagents (sgRNAs and hCAS9_mRNA) were used as RNA molecules and were not plasmid-coded. In this way, we intended to reduce the likelihood for

off-target effects, as RNA-based reagents are only short-lived (Tycko *et al*, 2019; Doench *et al*, 2016) in contrast to plasmid-coded reagents.

The corresponding verified mutated sequences were as follows:

D1563/1565/1570A

(5')gtcctttgacattgaggcctccttccccatggagtccatgttgacagtggccgtgtacgCctgggCtctggtgggcacAgCtgaTct catcggagaaaccaagattgacctggaaaaccgcttctacagcaagcatcgcgccacctgcggcatcgcacagacctattccat(3')

The sequence shown covers bp 4736-4933 (Exon38) of GenBank Acc. No. NM_031875, *Mus musculus* otoferlin (*Otof*), transcript variant 2, mRNA. Underlined, antisense KI specific primer site; red, D1563/1565/1570A missense mutation; blue, silent mutation.

I1573T

(5')gtcctttgacattgaggcctccttccccatggagtccatgttgacagtggccgtgtacgactgggatctggtgggcactgatgaTctca CcggCgaaaccaagattgacctggaaaaccgcttctacagcaagcaCcgcgcAacAtgcggcatcgcacagacctattccat(3')

The sequence shown covers bp 4736-4933 (Exon38) of GenBank Acc. No. NM_031875, *Mus musculus* otoferlin (*Otof*), transcript variant 2, mRNA. Underlined, antisense KI specific primer site; red, I1573T missense mutation; blue, silent mutation.

Successful insertion of *Otof*$^{D1563/1565/1570A}$ and *Otof*^{I1573T} was confirmed by genotyping according to standard PCR methods using DNA isolated from tail biopsies (using the Genomic DNA Isolation Kit for Tissue and Cells according to the manufacturer's protocol; Nexttec, Hilgertshausen, Germany). For quality control, all samples were analyzed on a 1% agarose gel. PCR products were analyzed by fragment analyses on a 3730XL-DNA-Analyzer (Applied Biosystems, now Life Technologies, Darmstadt, Germany). Genotyping primers were synthesized in house, sequence and fragment sizes are as follows:

Primer name	Sequence
36356	5'-ACTCCTTGCCCATCTCAAGC -3'
37212	5'-GGTTTCTCCGATGAGATCAGCT -3'
37213	5'-GTCAGGTTTCTCCGATGAGGTCATCA -3'
36532	5'-GTGCTCTTGGTTTCTCCGATGAGG -3'
36533	5'-TCTTGGTTTCGCCGGTGAGA -3'

Otof$^{D1563/1565/1570A}$

36356_37213_155bp = OTOTDA_WT_155

36356_37212_151bp = OTOTDA_KI_151

Otof^{I1573T}

36356_36532_159bp = I1573T_WT_159

36356_36533_155bp = I1573T_KI_155

ABR, DPOAE, and single unit recordings

Recordings of ABR, DPOAE, as well as single unit recordings from the auditory nerve in mice were performed largely as previously described (Jing *et al*, 2013). DPOAE were recorded using Tucker Davis Technologies (TDT) System III and custom Matlab software, TDT EC1 speakers, a Sennheiser MKE-2 microphone and Terratec DMX Fire or UAC zoom-2 microphone preamplifier.

Otof$^{TDA/TDA}$ mice were anesthetized by i.p. injection of a combination of urethane (1.32g/kg), xylazine (5mg/kg), and buprenorphine (0.1mg/kg). We then recorded ABRs using TDT System III, a free field JBL 2402 speaker, a custom amplifier (2500 times), and custom Matlab software. We applied tone bursts of 12 kHz at 80 dB and clicks at 80 and 100 dB for at least 2x1000 times and used digital bandpass filtering between 300 and 3000 Hz and averaging to obtain the ABR. Thereafter, we immediately turned to auditory nerve fiber recordings, using TDT System III, a ScanSpeak speaker (Avisoft), an ELC-03XS amplifier (NPI electronics), and custom Matlab software. As search stimulus, we used 50 ms 90 dB tone bursts at stimulus rates of 2 or 5 Hz.

Otof$^{I1573T/I1573T}$ mice were anesthetized by i.p. injection of a combination of ketamine (125 mg/kg) and xylazine (2.5 mg/kg). To record ABRs, signals between subcutaneous needle electrodes at the vertex and mastoid were amplified 50,000 times, bandpass filtered between 400 and 4,000 Hz (Neuroamp), and averaged at least 2x1,300 times for each stimulus type using TDT System II and BioSig software (TDT). Stimuli included tone bursts of varying frequencies at up to 80 dB and clicks at up to 120 dB.

In all in vivo experiments, the core temperature was maintained constant at 37°C using a heat blanket (Hugo Sachs Elektronik–Harvard Apparatus) or a custom-designed heat-plate. All stimuli were calibrated using a ¼ inch Bruel and Kjaer D 4039 microphone.

Patch-clamp

Apical turns of the organs of Corti from 2-week old mice were isolated in ice-cold HEPES Hank's solution containing (in mM): 5.26 KCl, 141.7 NaCl, 0.5 $MgSO_4.7H_2O$, 10 HEPES (4-(2-hydroxyethyl)-1-piperazineethane-sulfonic acid), 1 $MgCl_2$, 11.1 D-glucose and 3.42 L-glutamine, pH adjusted to around 7.2, osmolality ~300 mOsm/kg. The recording chamber was

perfused with modified Ringer's solution containing (in mM): 2.8 KCl, 111 NaCl, 35 TEA-Cl (tetraethylammonium chloride), 10 HEPES, 1 CsCl, 1 $MgCl_2$, 11.1 D-glucose, 2 $CaCl_2$ (1.3 $CaCl_2$ was used for *Otof*$^{I1573T/I1573T}$ mice to more closely approximate the physiological Ca^{2+} concentration), pH adjusted to around 7.2 and osmolality ~300 mOsm/kg. Cleaning of the tissue was performed to make the IHCs accessible for patch clamp by removing the tectorial membrane and neighboring cells. The clean exposed basolateral surface of IHCs was patch-clamped in perforated patch configuration using an EPC-10 amplifier (HEKA Electronics, Germany) controlled by *Patchmaster* software at room temperature, as described previously (Moser & Beutner, 2000). The pipette solution contained (in mM): 137 Cs-gluconate, 10 TEA-Cl, 10 4-aminopyridine, 10 HEPES, 1 $MgCl_2$, and 300 µg/ml of amphotericin B, pH adjusted to 7.2 using HCl and osmolality ~290 mOsm/kg. Cells were kept at a holding potential of -87 mV. All voltages were corrected for liquid junction potential (17 mV) offline. Currents were leak corrected using a p/10 protocol. Recordings were discarded when the leak current exceeded -50 pA, series resistance exceeded 30 MΩ, or Ca^{2+} current rundown exceeded 25%. Current-voltage relationships (IVs) were recorded once the access resistance dropped below 30 MΩ, by applying increasing 10 ms long step-depolarization pulses of voltage ranging from -87 mV to 65 mV, in steps of 5 mV. Exocytosis measurements were performed by measuring increments in membrane capacitance (ΔC_m) using the Lindau-Neher technique (Lindau & Neher, 1988). ΔC_m was recorded by stimulating the cells at the potential for maximal Ca^{2+} influx (-17 mV) for variable durations. Successive stimuli were acquired at an interval of 10-90 s. Each protocol was sequentially applied two to three times and only IHCs with reproducible measurements were included. For analysis, capacitance traces were averaged over 400 ms before and after the depolarization (skipping the first 60 ms after the end of depolarization). The traces were subjected to 5 or 10 pass binomial smoothing using Igor Pro 6 (WaveMetrics Inc., Lake Oswego, USA) for display. For dialysis patch-clamp experiments, the pipette solution contained (in mM): 137 Cs-gluconate, 10 TEA-Cl, 10 4-aminopyridine, 10 HEPES, 1 $MgCl_2$, 10 $CaCl_2$, 2 ATP, and 0.3 GTP, pH adjusted to 7.2 using HCl and osmolality ~290 mOsm/kg. ΔC_m data were recorded immediately after the cell membrane was ruptured. During recordings, the cells were kept at a holding potential of -87 mV. All recordings were performed in the same sequence for better comparability with previous data.

Protein purification

The Twin-Strep-tagged *Mus musculus* wild-type and TDA-otoferlin cDNA (pFastBac1 vector) was expressed in insect cells using the baculovirus expression system (Hitchman *et al*, 2009). Cells were lysed with Fos-Choline-12 (1 % w/v, Biozym) in presence of DNAse and protease

inhibitors (complete EDTA free protease inhibitors, Roche) in 20 mM HEPES, 500 mM KCl, 10% Sucrose, 5 mM DTT (pH-7.3), for 1 h at 4 °C. After removal of cell debris (250,000 x g for 30 min at 4 °C) the supernatant was submitted to affinity purification with Streptactin XT-4 Flow resin (IBA Life sciences) followed by anion exchange chromatography. The proteins were concentrated, flash frozen and stored at -80 °C prior to downstream analysis.

Nano DSF

A Prometheus NT.48 instrument (NanoTemper Technologies, Munich, Germany) was used to determine the melting temperatures. The capillaries were filled with 10 µl sample, placed on the sample holder, and each concentration was measured in duplicates. A temperature gradient of 0.5 $°C \cdot min^{-1}$ from 20 to 95 °C was applied. The laser power was adjusted to obtain a minimum of 2000 counts for each sample and the intrinsic protein fluorescence at 330 and 350 nm was recorded.

Immunohistochemistry and confocal microscopy

Mice (2-week-old, 4-week-old, and 6- to 8-week-old mice) were deeply anesthetized with CO_2 and sacrificed by decapitation for immediate dissection of the cochleae in ice-cold PBS. Fixation was performed by perfusing the cochleae with 4% formaldehyde (in PBS) for 60 min on ice, while for the labelling of synapses, a shorter fixation of 30 min was performed. The organs of Corti were dissected and washed briefly in PBS at room temperature. Blocking and permeabilization of the tissue was performed with GSDB (goat serum dilution buffer: 16% normal goat serum, 450 mM NaCl, 0.3% Triton X100, 20 mM phosphate buffer, pH ~7.4) for 1 h at room temperature. Samples were then incubated with primary antibodies (diluted in GSDB) overnight at 4°C and were washed three times for 10 min in wash buffer (450 mM NaCl, 0.3% Triton X 100, 20 mM phosphate buffer, pH ~ 7.4). This was followed by incubation with secondary antibodies (diluted in GSDB) for 1 h in a light-protected wet chamber. Finally, the samples were washed three times for 10 min in wash buffer before mounting onto glass slides with a drop of fluorescence mounting medium (Mowiol 4-88, Carl Roth, Karlsruhe, Germany) and covered with thin glass coverslips. Images were acquired in confocal/STED mode using an Abberior Instruments Expert Line STED microscope (Abberior Instruments GmbH, Göttingen, Germany). We employed lasers at 488, 561, and 633 nm for excitation. 1.4 NA 100X or 0.8 NA 20X oil immersion objectives were used. Confocal stacks were acquired using Imspector Software (Abberior Instruments GmbH, Göttingen, Germany; pixel size = 70 X 70 nm in xy, 200 nm in z). The acquired images were z-projected with NIH ImageJ software and adjusted for brightness and contrast. Organs of Corti from both mutant mice and

corresponding WT mice were always processed in parallel using identical staining protocols, laser excitation powers and microscope settings. Images were acquired and analyzed without a priori knowledge of the genotype.

Antibody	Host species	Company	Dilution	Identifier
**Primary antibody**				
Anti-N-otoferlin	Mouse (monoclonal IgG1)	Abcam	1:300	ab53233
Anti-C-otoferlin (1215)	Rabbit		1:300	
Anti-parvalbumin	Chicken (polyclonal)	Synaptic Systems	1:300	195006
Anti-ctbp2 (ribeye)	Mouse (monoclonal IgG1)	BD Biosciences	1:300	612044
Anti-homer1	Rabbit (polyclonal)	Synaptic Systems	1:500	160002
Anti-Vglut3	Rabbit (polyclonal)	Synaptic Systems	1:300	135203
Anti-GM130	Mouse (monoclonal IgG1)	BD Biosciences	1:200	610822
**Secondary antibody**				
STAR580 conjugated anti-mouse	Goat (polyclonal)	Abberior	1:200	2-0002-005-1
STAR635p conjugated anti-rabbit	Goat (polyclonal)	Abberior	1:200	2-0012-007-2
Alexa Fluor 488 conjugated anti-chicken	Goat (polyclonal)	Invitrogen	1:200	A11039
Alexa Fluor 647 conjugated anti-rabbit	Goat (polyclonal)	Invitrogen	1:200	A21244
Alexa Fluor 488 conjugated anti-rabbit	Goat (polyclonal)	Invitrogen	1:200	A11008

Conventional embedding for electron microscopy

Conventional embedding was performed as previously published (Wong *et al*, 2014; Michanski *et al*, 2019). Cochleae from three $Otof^{TDA/TDA}$ and one $Otof^{+/+}$ mice at P15 were dissected as described above. Subsequently, the organs of Corti were fixed immediately after dissection with 4% paraformaldehyde (0335.1, Carl Roth, Germany) and 0.5% glutaraldehyde (G7651, Sigma, Germany) in PBS (P4417, Sigma, Germany; pH 7.4) for 1 h on ice followed by a second fixation step overnight with 2% glutaraldehyde in 0.1 M sodium cacodylate buffer (v/v, pH 7.2) at 4°C. Next, specimens were washed in 0.1 M sodium cacodylate buffer and treated with 1% osmium tetroxide (75632, Sigma, Germany; v/v in 0.1 M sodium cacodylate buffer) for 1 h on ice followed by further sodium cacodylate buffer and distilled water washing steps. After the *en bloc* staining with 1% uranyl acetate (8473, Merck, Germany; v/v in distilled water) for 1 h on ice, samples were briefly washed in distilled water, dehydrated in an ascending concentration series of ethanol (30%, 50%, 70%, 95% and 100%), infiltrated and embedded in epoxy resin (R1140, AGAR-100, Plano, Germany) for at least 48 h at 70°C for final polymerization.

Ultrathin-sectioning and post-staining

The polymerized blocks were trimmed into a pyramidal shape to remove excess resin and 70 nm ultrathin sections were cut with a 35° diamond knife (Diatome AG, Biel, Switzerland) using an EM UC7 (Leica Microsystems, Wetzlar, Germany) ultramicrotome. Ultrathin sections were collected on 1% formvar-coated copper slot grids (Athene, 3.05 mm diameter, 1 x 2 mm;

G2500C, Plano). Post-staining was performed using UranyLess (22409, EMS, Hatfield, PA) for 20 min followed by several brief washing steps with distilled water.

Transmission electron microscopy

Electron micrographs were acquired at an 80 kV JEM1011 transmission electron microscope (JEOL) equipped with a Gatan Orius 1200A camera (GATAN GmbH, using the Digital Micrograph software package) at 12,000-x magnification.

Analysis of random sections

In random sections analysis, as previously described (Jean *et al*, 2018), MP-SVs were identified as the first-row of vesicles that fall into a distance of 25 nm from the AZ membrane and 80 nm from the presynaptic density (PD), while RA-SVs were identified as being in the first-row of vesicles around the ribbon with a maximum distance of 80 nm from its surface.

Quantitative PCR

Total RNA was extracted from mouse cochleae using the Monarch Total RNA Miniprep Kit (New England Biolabs) and the cDNA was synthesized using SuperScript III cDNA synthesis kit (Invitrogen, Thermo Fischer Scientific) with random Hexamer and Oligo(dT) 20 primers (Invitrogen, Thermo Fischer Scientific). Relative abundances were assessed by real-time quantitative PCR performed using GoTaq qPCR mix (Promega) according to the manufacturer's instructions. Primers were specific for *Otof* (Otof-F: 5'-GCTTCATCTACCGACCTCCAG-3', Otof-R: 5'-TTCTTCTTCCTTTCCTTCTCATCTTC-3'), hypoxanthine phosphoribosyltransferase (*Hprt*) (Hprt-F: 5'-CCTCCTCAGACCGCTTTTT-3', Hprt-R: 5'-AACCTGGTTCATCATCGCTAA-3') and Ribosomal protein 13 (*Rps13*) (Rps13-F: 5'-CGAAAGCACCTTGAGAGGAA-3', Rps13-R: 5'-TTCCAATTAGGTGGGAGCAC-3'). Mean of *Otof* mRNA abundance was normalized to the mean of *Rps13* and *Hprt* mRNA abundance.

Statistical analysis

Data are mainly presented as box and whisker plots presenting median, lower/upper quartiles and 10–90th percentiles with individual data points overlaid or bar plots with mean ± SEM. Data were analysed using Excel and Igor Pro 6 and 7 (WaveMetrics Inc.). Using Igor Pro, normality of data was assessed with the Jarque-Bera test or the Wald-Wolfowitz test and equality of variances in normally distributed data was assessed with the F-test. Differences between two groups were evaluated for significant differences using the two-tailed unpaired

Student's *t*-test, or, when not normally distributed and/or variance was unequal, the unpaired two-tailed Mann–Whitney Wilcoxon test. Non-significant differences between samples are indicated as *n.s.*, significant differences are indicated as $*p < 0.05$, $**p < 0.01$, $***p < 0.001$.

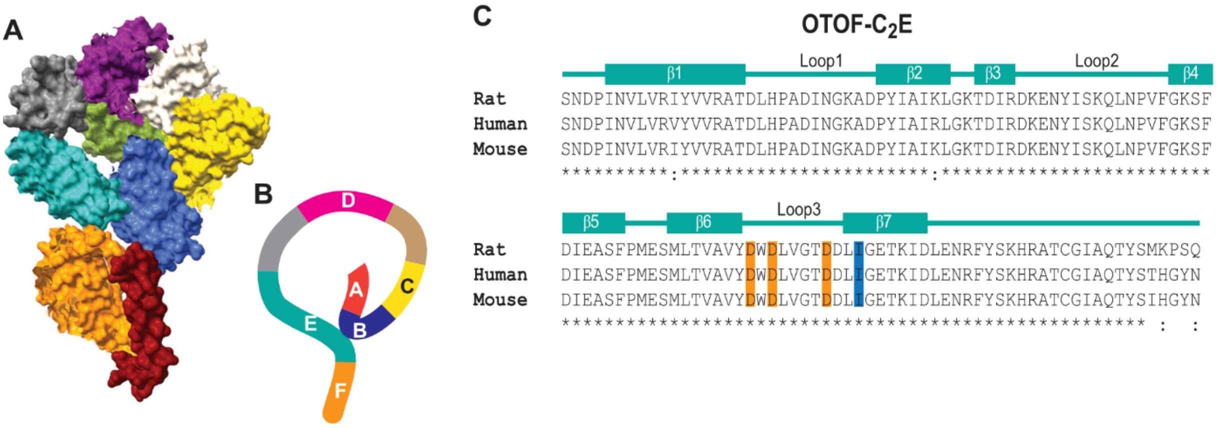

Figure S1. Predicted structure of otoferlin and amino acids of C_2E targeted by mutagenesis. Related to Figure 1. (**A-B**) AlphaFold2-predicted overall structure of the otoferlin protein in surface representation (A) and a simplified schematic drawing (B). (**C**) Structure-based sequence alignment of otoferlin C_2E domain among species: conservation of the 3 aspartates substituted by alanine in *Otof*$^{TDA/TDA}$ (orange highlights) and isoleucine 1573 (blue highlight).

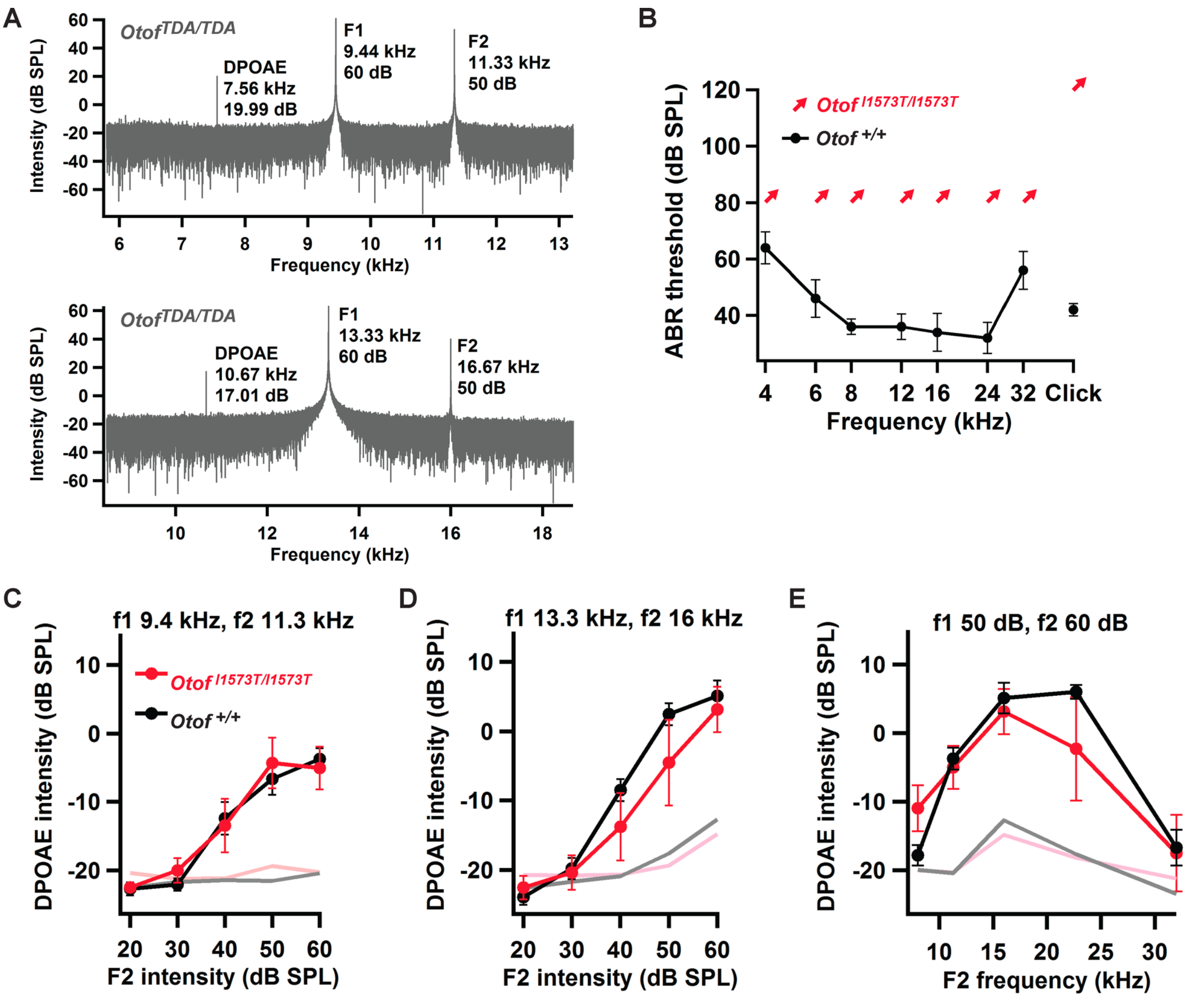

Figure S2. Mutation of the C2E domain causes synaptopathic hearing impairment in mice. Related to Figure 1. (**A**) *Otof$^{TDA/TDA}$* mice showed DPOAE (examples at two frequency/intensity combinations of probe tones). (**B**) *Otof$^{+/+}$* mice had normal ABR thresholds (N = 5 mice), whereas in *Otof$^{I1573T/I1573T}$* mice ABRs were never observed up to the maximal loudspeaker output of 80 dB for tone bursts and 120 dB for click stimuli (N = 5 mice). (**C-E**) *Otof$^{I1573T/I1573T}$* mice showed normal DPOAE, no significant difference was observed between wild-type (WT; N = 5 mice) and mutant (N = 5 mice) mice. DPOAE growth functions for two probe tone frequency pairs (C, D). DPOAE amplitude as a function of probe tone frequency (E).

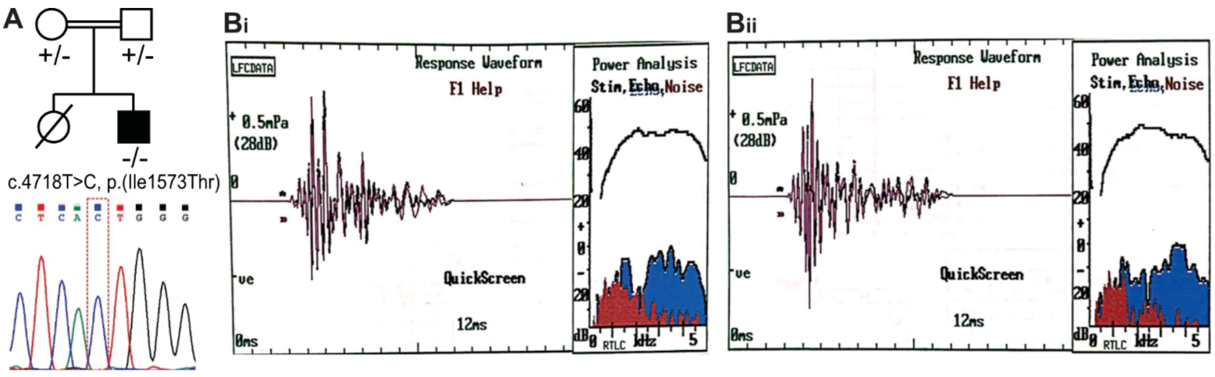

Figure S3. A human mutation of the C$_2$E domain causes synaptopathic hearing impairment. Related to Figure 1.

(**A**) Pedigree of the patient with Sanger sequencing electropherogram below showing the homozygous c.4718T>C, p.(Ile1573Thr) variant. Symbols: - represents the variant, + represents the reference allele. (**B**) Transient evoked otoacoustic emissions of the *OTOF*^{I1573T} patient at approximately 5 years of age: right ear (B$_i$) and left ear (B$_{ii}$).

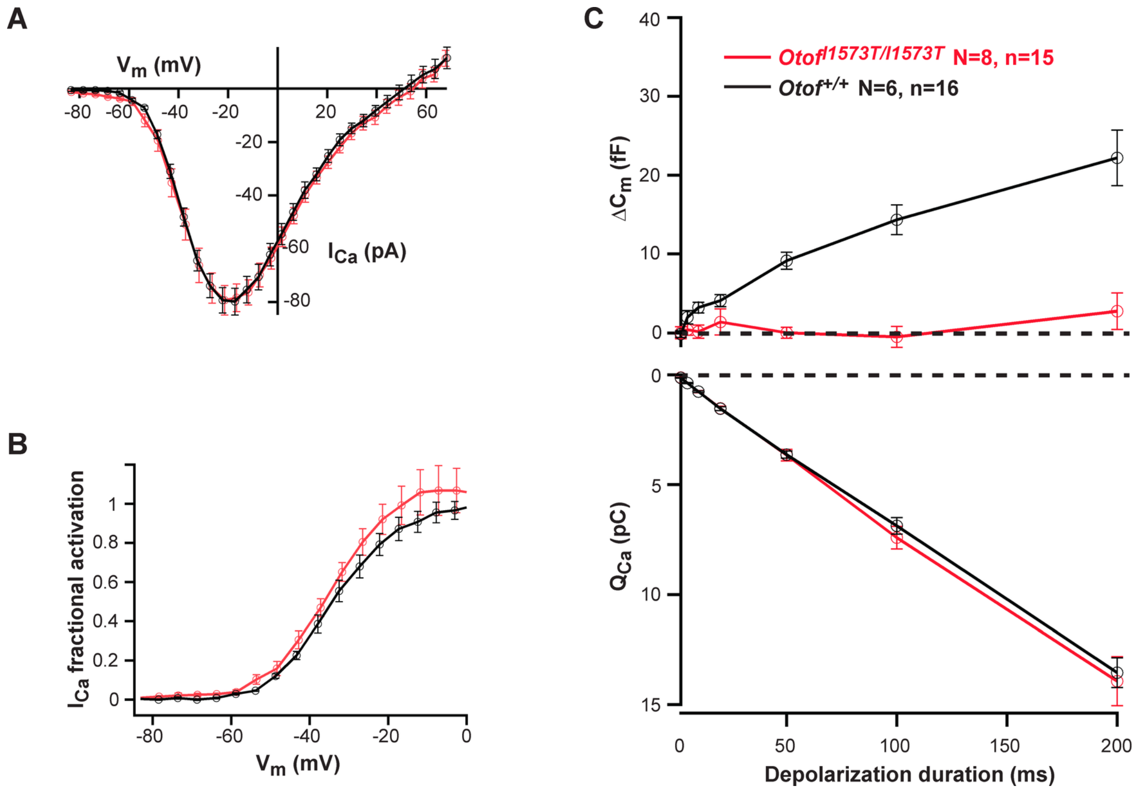

Figure S4. Ca^{2+} influx triggered exocytosis is largely abolished in *Otof*$^{I1573T/I1573T}$ IHCs. Related to Figure 1. (**A, B**) No significant difference in voltage-dependent Ca^{2+} currents (A) and fractional activation of I_{Ca} channels (B) between *Otof*$^{I1573T/I1573T}$ and *Otof*$^{+/+}$ mice (mean ± SEM, n = 15 *Otof*$^{I1573T/I1573T}$ IHCs from N = 8 *Otof*$^{I1573T/I1573T}$mice, red; n = 16 *Otof*$^{+/+}$ IHCs from N = 6 *Otof*$^{+/+}$ mice, black). (**C**) Kinetics of exocytosis (top) and corresponding Ca^{2+} current integrals (bottom) of *Otof*$^{I1573T/I1573T}$ and *Otof*$^{+/+}$ IHCs (mean ± SEM). Data were acquired in 1.3 mM extracellular Ca^{2+}.

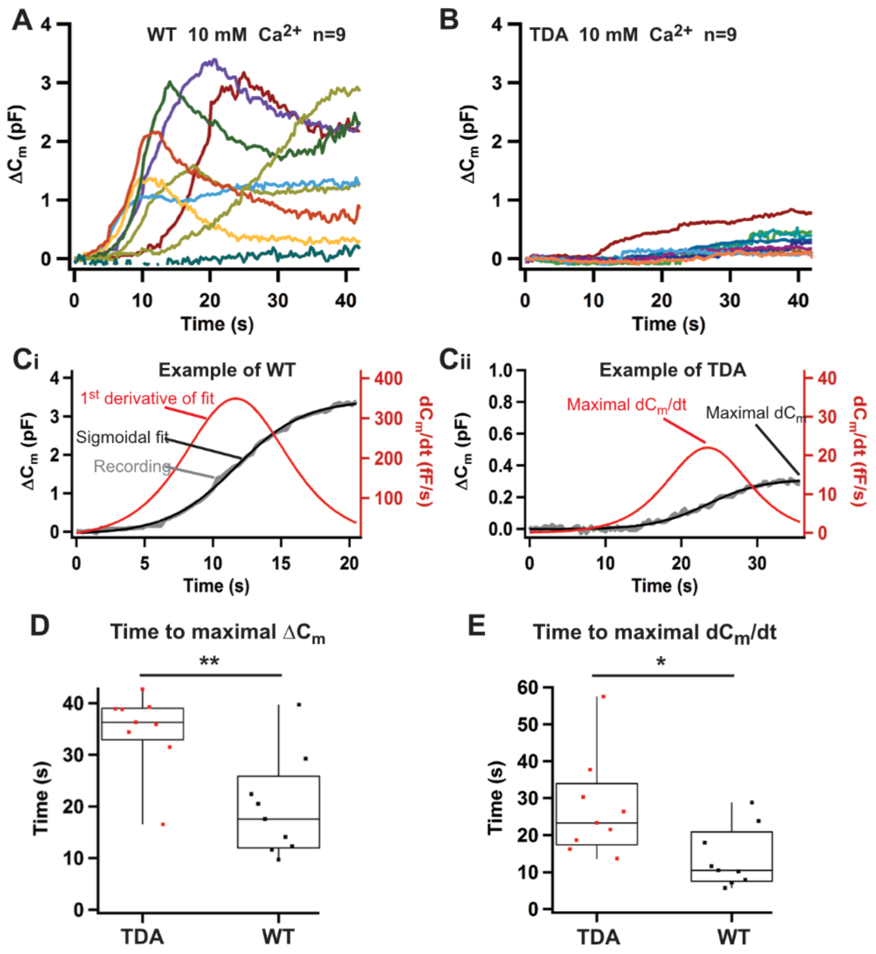

Figure S5. Partial restoration of exocytosis in *Otof*$^{TDA/TDA}$ IHCs upon Ca^{2+} dialysis. Related to Figure 1.

(**A-B**) Membrane capacitance recordings with a high concentration of Ca^{2+} (10 mM) in the pipette revealed exocytosis in both *Otof*$^{+/+}$ (A) and *Otof*$^{TDA/TDA}$ (B) mouse IHCs. (**C**) Examples of the analysis of single capacitance traces from *Otof*$^{+/+}$ (Ci) and *Otof*$^{TDA/TDA}$ IHCs (Cii). Every recording is fitted with a sigmoid function, the fit was then differentiated to obtain the rate of capacitance change (dC_m/dt). (**D, E**) Maximal ΔC_m and maximal dC_m/dt were calculated from the fit and its first derivative, respectively. Time to maximal capacitance change (D), and time to maximal dC_m/dt (E) of *Otof*$^{TDA/TDA}$ and *Otof*$^{+/+}$ mouse IHCs.

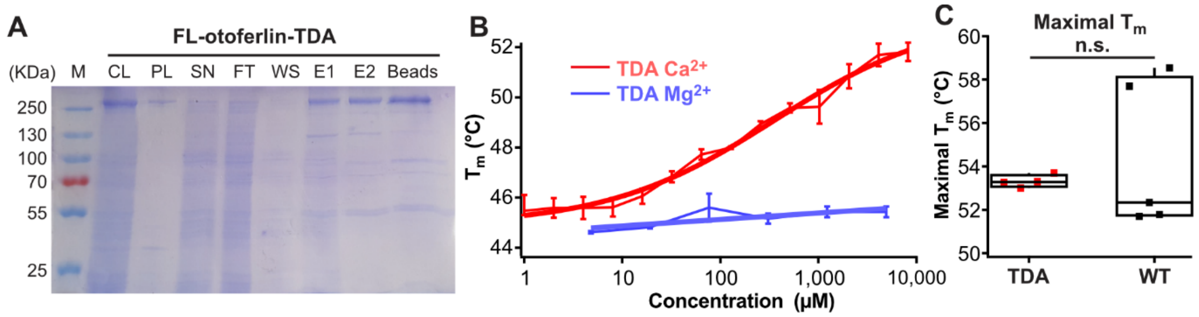

Figure S6. TDA-otoferlin has a lower apparent affinity to Ca^{2+}. Related to Figure 1.

(**A**) Coomassie-stained SDS-PAGE gel showing affinity-purified full-length (FL) TDA-otoferlin from insect cells. Proteins were further used for nanoDSF measurements. M: marker, CL: cell lysate, PL: pellet, SN: supernatant, FT: flowthrough, WS: washing, E: elution. (**B-C**) Ca^{2+} binding by TDA-otoferlin increases its thermal stability, quantified as the melting temperature (Tm) in a dose-dependent manner as shown by nanoDSF. Mg^{2+} was used as a control ion and did not affect Tm suggesting a lack of binding (B). Maximal Tm calculated from the fitted curves was not significantly different between TDA- and WT-otoferlin, arguing against a decreased thermal stability of TDA-otoferlin (C).

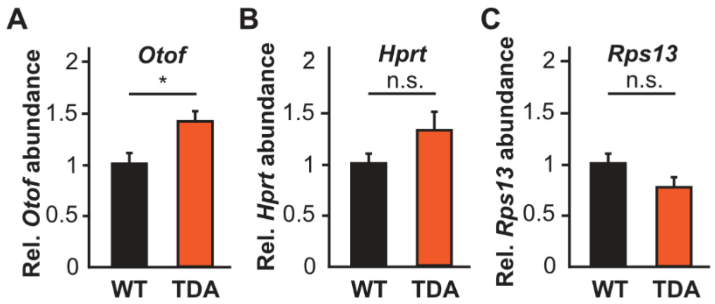

Figure S7. Reduction of otoferlin protein in *Otof*$^{TDA/TDA}$ IHC levels despite slightly elevated mRNA levels in *Otof*$^{TDA/TDA}$. Related to Figure 2

(**A**) qPCR analysis revealed maintained *Otof* mRNA levels in IHCs of *Otof*$^{TDA/TDA}$ mice (N = 3). (**B, C**) *Hprt* (B) and *Rps13* (C) were sampled as housekeeping genes: no significant differences were detected between *Otof*$^{TDA/TDA}$ and *Otof*$^{+/+}$ control mice ($N = 4$). (mean ± SEM, *$p < 0.05$).

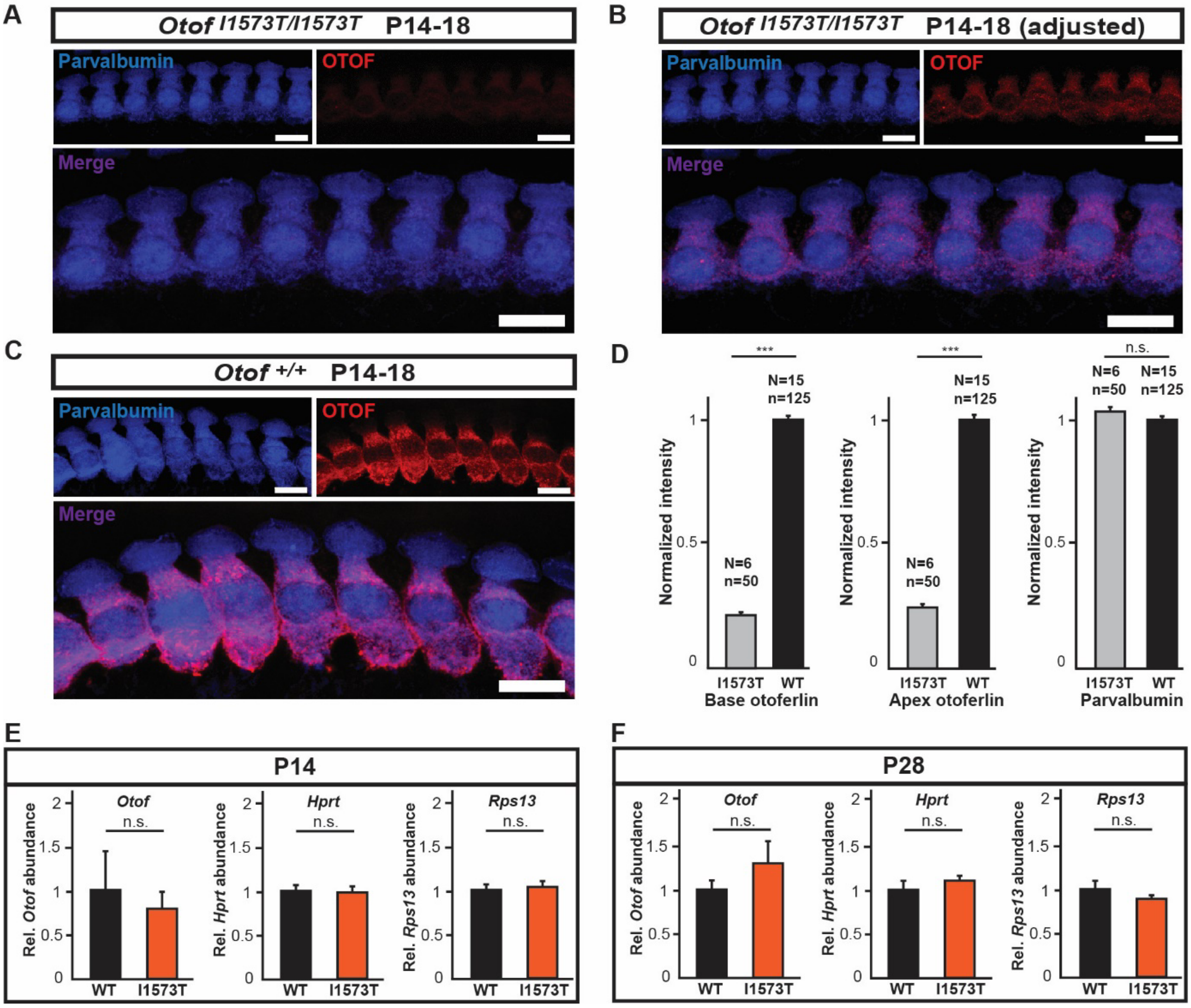

Figure S8. Reduction of otoferlin protein levels despite normal mRNA levels in *Otof*$^{I1573T/I1573T}$. Related to Figure 2. (**A-C**) Maximum intensity projections of confocal stacks of postnatal week 2-3 mouse IHCs of *Otof*$^{I1573T/I1573T}$ (A, B) and *Otof*$^{+/+}$ mice (C) following immunolabeling for parvalbumin (blue) and otoferlin (red). (A) and (C) are acquired at identical gain settings. (B) is the same as (A), but after setting a higher gain to show a better signal of otoferlin. Scale bar, 10 µm. (**D**) Otoferlin protein levels were largely reduced in *Otof*$^{I1573T/I1573T}$ IHCs (N represents numbers of animals and n represents numbers of cells) compared to control *Otof*$^{+/+}$ IHCs (mean ± SEM, ***p < 0.001). (**E, F**) qPCR results showed that *Otof* mRNA level in *Otof*$^{I1573T/I1573T}$ IHCs ($N = 3$) is not different from that in *Otof*$^{+/+}$ IHCs ($N = 4$) for postnatal day 14 (P14) animals (E). Housekeeping genes *Hprt* and *Rps13* were checked in parallel as quality control, no significant difference was detected between *Otof*$^{I1573T/I1573T}$ and *Otof*$^{+/+}$ mice (mean ± SEM). The same for P28 animals (F).

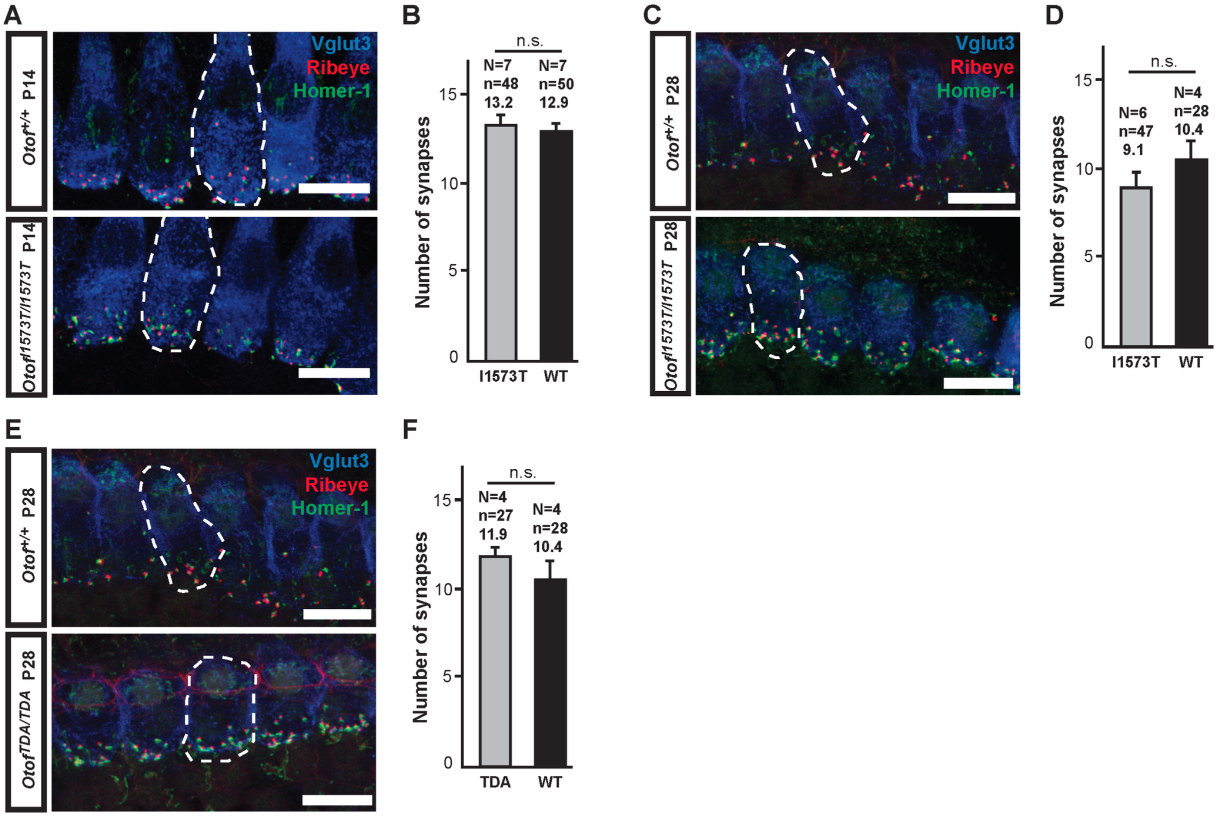

Figure S9. The number of ribbon synapses in *Otof*$^{TDA/TDA}$ and *Otof*$^{I1573T/I1573T}$ IHCs remain unchanged. Related to Figure 2. (**A**) Maximum-intensity projections of confocal stacks of P14 mouse IHCs of *Otof*$^{+/+}$ and *Otof*$^{I1573T/I1573T}$ IHCs following immunolabeling for Vglut3 (blue), Ribeye (red) and Homer-1 (green). Only juxtaposed red and green dots are counted as ribbon synapses. Scale bar, 10 µm. (**B**) The numbers of ribbon synapses per IHC are similar between *Otof*$^{I1573T/I1573T}$ and *Otof*$^{+/+}$ mice at P14. (**C**) Same as (A) for P28 mouse IHCs. Scale bar, 10 µm. (**D**) The numbers of ribbon synapses per IHC are similar between *Otof*$^{I1573T/I1573T}$ and *Otof*$^{+/+}$ mice at P28. (**E**) Same as (B) but for IHCs from *Otof*$^{+/+}$ and *Otof*$^{TDA/TDA}$ mice. Scale bar, 10 µm. (**F**) The numbers of ribbon synapses per IHC are similar between *Otof*$^{TDA/TDA}$ and *Otof*$^{+/+}$ mice at P28.

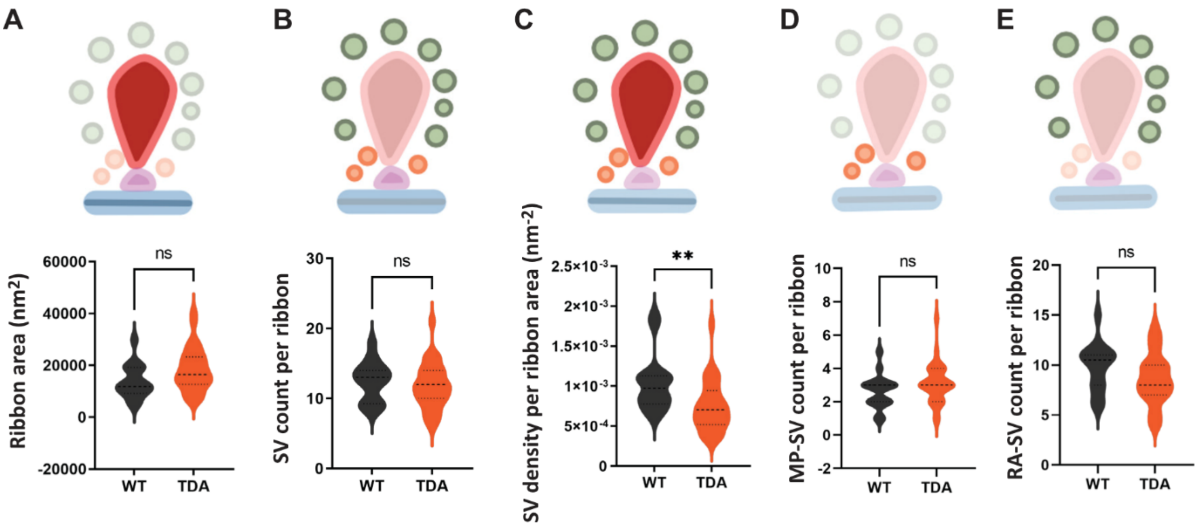

Figure S10. Largely unaltered ultrastructure of ribbon synapses in *Otof*$^{TDA/TDA}$ IHCs. Related to Figure 2. (**A-E**) Random section analysis of ribbon area (**A**), total SV count per ribbon (**B**), SV density determined per ribbon area (**C**), MP-SV count per ribbon (**D**), and RA-SV count per ribbon (**E**). *Otof*$^{TDA/TDA}$: $N = 3$ mice, $n = 31$ ribbons. *Otof*$^{+/+}$: $N = 1$ mouse, $n = 16$ ribbons. $^{**}p < 0.01$.

Extended Discussion

In the present study, we used targeted mutagenesis to examine the role of the C_2E domains of otoferlin in IHC presynaptic function and hearing. Genetic perturbation focused on the top loops of the C_2E domains that are involved in Ca^{2+} binding. Moreover, we studied the human *OTOF*^{I1573T} missense mutation in a pediatric case and in corresponding knock-in mice (*Otof*$^{I1573T/I1573T}$). ABRs were abolished in the human *OTOF*^{I1573T} case and in the mouse mutants despite intact cochlear amplification by OHCs, as assessed by otoacoustic emissions. Detailed molecular and cellular analysis of the novel mouse mutants revealed a lack of Ca^{2+} influx-triggered IHC exocytosis despite considerable expression of otoferlin remaining in IHCs of both lines.

The reduced Ca^{2+} affinity of TDA-otoferlin, found *in vitro,* might place the Ca^{2+} dependence of exocytosis in *Otof*$^{TDA/TDA}$ IHCs outside the range of [Ca^{2+}] achieved by voltage-gated Ca^{2+} influx (maximally ~40 µM in the Ca^{2+} microdomain control (Neef *et al*, 2018) and likely 100-200 µM in the Ca^{2+} nanodomain control (Roberts, 1993; Wong *et al*, 2014)) and Ca^{2+} uncaging (up to ~100 µM (Beutner *et al*, 2001)). The interpretation of the results obtained from *Otof*$^{I1573T/I1573T}$ IHCs is more challenging. The establishment of a protocol for purifying sufficient quantities of high-quality recombinant full-length otoferlin from insect cells will help further *in vitro* analysis of mutant otoferlin such I1573T-otoferlin. This will assess Ca^{2+} binding e.g. by isothermal titration calorimetry in the presence and absence of phospholipids, and structure, e.g. by cryo-electron microscopy. Such future studies of human *OTOF* missense mutations, combining analyses of recombinant protein with multiscale analyses of auditory function in otoferlin mutant mice, will be instrumental for understanding the precise disease mechanism(s). Finally, combinatorial mutagenesis experiments targeting Ca^{2+} binding sites of several C_2 domains should be pursued to dissect the Ca^{2+} sensing functions of otoferlin in SV fusion and replenishment (Michalski *et al*, 2017; Pangrsic *et al*, 2010).

Although CRISPR/Cas9 gene editing has massively expedited the generation of mouse mutants, it will be important, e.g. for rapid functional analyses, to optimize viral gene transfer for efficient expression of selected otoferlin mutants using *Otof*$^{-/-}$ IHCs. Expression of full-length (Al-Moyed *et al*, 2019; Akil *et al*, 2019; Rankovic *et al*, 2021) and partial deletion constructs of otoferlin (mini-otoferlins (Tertrais *et al*, 2019)) via AAV mediated gene transfer partially restores function in *Otof*$^{-/-}$ IHCs. However, achieving WT levels of otoferlin, especially of mutant variants, will likely remain difficult to achieve. As mentioned above, SV fusion in IHCs tolerates lower levels of otoferlin than SV replenishment, restoration of which seems to be required for restoring auditory signaling (Al-Moyed *et al*, 2019). Interestingly, while

expression of full-length otoferlin in *Otof*$^{-/-}$ IHCs led to partial restoration of ABRs in all three studies published (Al-Moyed *et al*, 2019; Akil *et al*, 2019; Rankovic *et al*, 2021), ABRs were not observed upon expression of mini-otoferlins (Tertrais *et al*, 2019). Unfortunately, the autaptic neural culture system, a workhorse in molecular synapse physiology, or chromaffin cells do not provide a work-around here as their synaptotagmin cannot be substituted by transgenic otoferlin (Reisinger *et al*, 2011). Analysis of afferent hair cell synapses in the lateral line organ of zebrafish provides a valuable option (Trapani & Nicolson, 2011; Sebe *et al*, 2017; Wong *et al*, 2019; Sheets *et al*, 2017) and has been successfully employed for studying otoferlin function (Chatterjee *et al*, 2015). Yet, intracellular recordings from lateral line hair cells are challenging (Olt *et al*, 2016). In conclusion, the search for an efficient cellular system for analyzing mutant otoferlin in mature IHCs remains an important objective for future studies.

References

Akil O, Dyka F, Calvet C, Emptoz A, Lahlou G, Nouaille S, Monvel JB de, Hardelin J-P, Hauswirth WW, Avan P, *et al* (2019) Dual AAV-mediated gene therapy restores hearing in a DFNB9 mouse model. *PNAS* 116: 4496–4501

Al-Moyed H, Cepeda AP, Jung S, Moser T, Kügler S & Reisinger E (2019) A dual-AAV approach restores fast exocytosis and partially rescues auditory function in deaf otoferlin knock-out mice. *EMBO Mol Med* 11: e9396

Baig SM, Koschak A, Lieb A, Gebhart M, Dafinger C, Nürnberg G, Ali A, Ahmad I, Sinnegger-Brauns MJ, Brandt N, *et al* (2011) Loss of Ca(v)1.3 (CACNA1D) function in a human channelopathy with bradycardia and congenital deafness. *Nat Neurosci* 14: 77–84

Becker L, Schnee ME, Niwa M, Sun W, Maxeiner S, Talaei S, Kachar B, Rutherford MA & Ricci AJ (2018) The presynaptic ribbon maintains vesicle populations at the hair cell afferent fiber synapse. *eLife Sciences* 7: e30241

Beurg M, Michalski N, Safieddine S, Bouleau Y, Schneggenburger R, Chapman ER, Petit C & Dulon D (2010) Control of exocytosis by synaptotagmins and otoferlin in auditory hair cells. *J Neurosci* 30: 13281–13290

Beutner D, Voets T, Neher E & Moser T (2001) Calcium Dependence of Exocytosis and Endocytosis at the Cochlear Inner Hair Cell Afferent Synapse. *Neuron* 29: 681–690

Buran BN, Strenzke N, Neef A, Gundelfinger ED, Moser T & Liberman MC (2010) Onset coding is degraded in auditory nerve fibers from mutant mice lacking synaptic ribbons. *J Neurosci* 30: 7587–7597

Calvet C, Peineau T, Benamer N, Cornille M, Lelli A, Plion B, Lahlou G, Fanchette J, Nouaille S, Monvel JB de, *et al* (2022) The SNARE protein SNAP-25 is required for normal exocytosis at auditory hair cell ribbon synapses. *iScience* 25

Chatterjee P, Padmanarayana M, Abdullah N, Holman CL, LaDu J, Tanguay RL & Johnson CP (2015) Otoferlin Deficiency in Zebrafish Results in Defects in Balance and Hearing: Rescue of the Balance and Hearing Phenotype with Full-Length and Truncated Forms of Mouse Otoferlin. *Mol Cell Biol* 35: 1043–1054

Doench JG, Fusi N, Sullender M, Hegde M, Vaimberg EW, Donovan KF, Smith I, Tothova Z, Wilen C, Orchard R, *et al* (2016) Optimized sgRNA design to maximize activity and minimize off-target effects of CRISPR-Cas9. *Nat Biotechnol* 34: 184–191

Golbek TW, Padmanarayana M, Roeters SJ, Weidner T, Johnson CP & Baio JE (2019) Otoferlin C2F Domain-Induced Changes in Membrane Structure Observed by Sum Frequency Generation. *Biophys J* 117: 1820–1830

Greene CC, McMillan PM, Barker SE, Kurnool P, Lomax MI, Burmeister M & Lesperance MM (2001) DFNA25, a novel locus for dominant nonsyndromic hereditary hearing impairment, maps to 12q21-24. *Am J Hum Genet* 68: 254–260

Gregory FD, Bryan KE, Pangrsic T, Calin-Jageman IE, Moser T & Lee A (2011) Harmonin inhibits presynaptic Cav1.3 Ca2+ channels in mouse inner hair cells. *Nat Neurosci* 14: 1109–1111

Hams N, Padmanarayana M, Qiu W & Johnson CP (2017) Otoferlin is a multivalent calcium-sensitive scaffold linking SNAREs and calcium channels. *Proc Natl Acad Sci USA* 114: 8023–8028

Helfmann S, Neumann P, Tittmann K, Moser T, Ficner R & Reisinger E (2011) The crystal structure of the C_2A domain of otoferlin reveals an unconventional top loop region. *J Mol Biol* 406: 479–490

Hitchman RB, Possee RD & King LA (2009) Baculovirus expression systems for recombinant protein production in insect cells. *Recent Pat Biotechnol* 3: 46–54

Jean P, Morena DL de la, Michanski S, Tobón LMJ, Chakrabarti R, Picher MM, Neef J, Jung S, Gültas M, Maxeiner S, *et al* (2018) The synaptic ribbon is critical for sound encoding at high rates and with temporal precision. *Elife* 7: e29275

Jing Z, Rutherford MA, Takago H, Frank T, Fejtova A, Khimich D, Moser T & Strenzke N (2013) Disruption of the presynaptic cytomatrix protein bassoon degrades ribbon anchorage, multiquantal release, and sound encoding at the hair cell afferent synapse. *J Neurosci* 33: 4456–4467

Johnson CP & Chapman ER (2010) Otoferlin is a calcium sensor that directly regulates SNARE-mediated membrane fusion. *J Cell Biol* 191: 187–197

Johnson SL, Franz C, Kuhn S, Furness DN, Ruttiger L, Munkner S, Rivolta MN, Seward EP, Herschman HR, Engel J, *et al* (2010) Synaptotagmin IV determines the linear Ca2+ dependence of vesicle fusion at auditory ribbon synapses. *Nat Neurosci* 13: 45–52

Johnson SL, Safieddine S, Mustapha M & Marcotti W (2019) Hair Cell Afferent Synapses: Function and Dysfunction. *Cold Spring Harb Perspect Med* 9

Jumper J, Evans R, Pritzel A, Green T, Figurnov M, Ronneberger O, Tunyasuvunakool K, Bates R, Žídek A, Potapenko A, *et al* (2021) Highly accurate protein structure prediction with AlphaFold. *Nature* 596: 583–589

Jung S, Oshima-Takago T, Chakrabarti R, Wong AB, Jing Z, Yamanbaeva G, Picher MM, Wojcik SM, Göttfert F, Predoehl F, *et al* (2015a) Rab3-interacting molecules 2α and 2β promote the abundance of voltage-gated CaV1.3 Ca2+ channels at hair cell active zones. *Proc Natl Acad Sci USA* 112: E3141–E3149

Jung SY, Maritzen T, Wichmann C, Jing ZZ, Neef A, Revelo NH, Al-Moyed H, Meese S, Wojcik SM, Panou I, *et al* (2015b) Disruption of adaptor protein 2 mu (AP-2 mu) in cochlear hair cells impairs vesicle reloading of synaptic release sites and hearing. *Embo J* 34: 2686–2702

Khimich D, Nouvian R, Pujol R, tom Dieck S, Egner A, Gundelfinger ED & Moser T (2005) Hair cell synaptic ribbons are essential for synchronous auditory signalling. *Nature* 434: 889–894

Kopanos C, Tsiolkas V, Kouris A, Chapple CE, Albarca Aguilera M, Meyer R & Massouras A (2019) VarSome: the human genomic variant search engine. *Bioinformatics* 35: 1978–1980

Krinner S, Butola T, Jung S, Wichmann C & Moser T (2017) RIM-Binding Protein 2 Promotes a Large Number of CaV1.3 Ca2+-Channels and Contributes to Fast Synaptic Vesicle Replenishment at Hair Cell Active Zones. *Front Cell Neurosci* 11: 334

Krinner S, Predoehl F, Burfeind D, Vogl C & Moser T (2021) RIM-Binding Proteins Are Required for Normal Sound-Encoding at Afferent Inner Hair Cell Synapses. *Front Mol Neurosci* 14: 651935

Lek A, Evesson FJ, Sutton RB, North KN & Cooper ST (2012) Ferlins: Regulators of Vesicle Fusion for Auditory Neurotransmission, Receptor Trafficking and Membrane Repair. *Traffic* 13: 185–194

Lindau M & Neher E (1988) Patch-clamp techniques for time-resolved capacitance measurements in single cells. *Pflügers Archiv European Journal of Physiology* 411: 137–146

McNeil PL & Kirchhausen T (2005) An emergency response team for membrane repair. *Nat Rev Mol Cell Biol* 6: 499–505

Michalski NA, Goutman JD, Auclair SM, Monvel JB de, Tertrais M, Emptoz A, Parrin A, Nouaille S, Guillon M, Sachse M, *et al* (2017) Otoferlin acts as a Ca2+ sensor for vesicle fusion and vesicle pool replenishment at auditory hair cell ribbon synapses. *eLife* 6: e31013

Michanski S, Smaluch K, Steyer AM, Chakrabarti R, Setz C, Oestreicher D, Fischer C, Möbius W, Moser T, Vogl C, *et al* (2019) Mapping developmental maturation of inner hair cell ribbon synapses in the apical mouse cochlea. *PNAS* 116: 6415–6424

Moser T (2020) Presynaptic Physiology of Cochlear Inner Hair Cells. In *The Senses: A Comprehensive Reference* pp 441–467. Elsevier

Moser T & Beutner D (2000) Kinetics of exocytosis and endocytosis at the cochlear inner hair cell afferent synapse of the mouse. *Proc Natl Acad Sci U S A* 97: 883–888

Moser T & Starr A (2016) Auditory neuropathy — neural and synaptic mechanisms. *Nat Rev Neurol* 12: 135–149

Moser T, Strenzke N, Meyer A, Lesinski-Schiedat A, Lenarz T, Beutner D, Foerst A, Lang-Roth R, Wedel H, Walger M, *et al* (2006) Diagnostik und Therapie der auditorischen Synaptopathie/Neuropathie. *HNO* 54: 833–841

Müller TM, Gierke K, Joachimsthaler A, Sticht H, Izsvák Z, Hamra FK, Fejtová A, Ackermann F, Garner CC, Kremers J, *et al* (2019) A multiple Piccolino-RIBEYE interaction supports plate-shaped synaptic ribbons in retinal neurons. *J Neurosci* 39: 2038–18

Neef J, Urban NT, Ohn T-L, Frank T, Jean P, Hell SW, Willig KI & Moser T (2018) Quantitative optical nanophysiology of Ca^{2+} signaling at inner hair cell active zones. *Nat Commun* 9: 290

Nouvian R, Neef J, Bulankina AV, Reisinger E, Pangršič T, Frank T, Sikorra S, Brose N, Binz T & Moser T (2011) Exocytosis at the hair cell ribbon synapse apparently operates without neuronal SNARE proteins. *Nat Neurosci* 14: 411–413

Ohn T-L, Rutherford MA, Jing Z, Jung S, Duque-Afonso CJ, Hoch G, Picher MM, Scharinger A, Strenzke N & Moser T (2016) Hair cells use active zones with different voltage dependence of Ca^{2+} influx to decompose sounds into complementary neural codes. *PNAS* 113: E4716–E4725

Olt J, Ordoobadi AJ, Marcotti W & Trapani JG (2016) Physiological recordings from the zebrafish lateral line. *Methods Cell Biol* 133: 253–279

Oza AM, DiStefano MT, Hemphill SE, Cushman BJ, Grant AR, Siegert RK, Shen J, Chapin A, Boczek NJ, Schimmenti LA, *et al* (2018) Expert specification of the ACMG/AMP variant interpretation guidelines for genetic hearing loss. *Hum Mutat* 39: 1593–1613

Pangrsic T, Lasarow L, Reuter K, Takago H, Schwander M, Riedel D, Frank T, Tarantino LM, Bailey JS, Strenzke N, *et al* (2010) Hearing requires otoferlin-dependent efficient replenishment of synaptic vesicles in hair cells. *Nat Neurosci* 13: 869–876

Pangršič T, Reisinger E & Moser T (2012) Otoferlin: a multi-C2 domain protein essential for hearing. *Trends Neurosci* 35: 671–680

Picher MM, Gehrt A, Meese S, Ivanovic A, Predoehl F, Jung S, Schrauwen I, Dragonetti AG, Colombo R, Camp GV, *et al* (2017a) Ca2+-binding protein 2 inhibits Ca2+-channel inactivation in mouse inner hair cells. *PNAS* 114: E1717–E1726

Picher MM, Oprişoreanu A-M, Jung S, Michel K, Schoch S & Moser T (2017b) Rab Interacting Molecules 2 and 3 Directly Interact with the Pore-Forming CaV1.3 Ca2+ Channel Subunit and Promote Its Membrane Expression. *Front Cell Neurosci* 11

Rankovic V, Vogl C, Dörje NM, Bahader I, Duque-Afonso CJ, Thirumalai A, Weber T, Kusch K, Strenzke N & Moser T (2021) Overloaded Adeno-Associated Virus as a Novel Gene Therapeutic Tool for Otoferlin-Related Deafness. *Front Mol Neurosci* 13

Reisinger E, Bresee C, Neef J, Nair R, Reuter K, Bulankina A, Nouvian R, Koch M, Bückers J, Kastrup L, *et al* (2011) Probing the functional equivalence of otoferlin and synaptotagmin 1 in exocytosis. *J Neurosci* 31: 4886–4895

Roberts WM (1993) Spatial calcium buffering in saccular hair cells. *Nature* 363: 74–76

Romanos J, Kimura L, Fávero ML, Izarra FAR, de Mello Auricchio MTB, Batissoco AC, Lezirovitz K, Abreu-Silva RS & Mingroni-Netto RC (2009) Novel OTOF mutations in Brazilian patients with auditory neuropathy. *J Hum Genet* 54: 382–385

Roux I, Safieddine S, Nouvian R, Grati M, Simmler M-C, Bahloul A, Perfettini I, Le Gall M, Rostaing P, Hamard G, *et al* (2006) Otoferlin, Defective in a Human Deafness Form, Is Essential for Exocytosis at the Auditory Ribbon Synapse. *Cell* 127: 277–289

Rutherford MA, Von Gersdorff H & Goutman JD (2021) Encoding sound in the cochlea: from receptor potential to afferent discharge. *The Journal of Physiology* 599: 2527–2557

Safieddine S, El-Amraoui A & Petit C (2012) The auditory hair cell ribbon synapse: from assembly to function. *Annu Rev Neurosci* 35: 509–528

Santarelli R, Del Castillo I, Cama E, Scimemi P & Starr A (2015) Audibility, speech perception and processing of temporal cues in ribbon synaptic disorders due to OTOF mutations. *Hear Res* 330: 200–212

Schrauwen I, Helfmann S, Inagaki A, Predoehl F, Tabatabaiefar MA, Picher MM, Sommen M, Seco CZ, Oostrik J, Kremer H, *et al* (2012) A Mutation in CABP2, Expressed in Cochlear Hair Cells, Causes Autosomal-Recessive Hearing Impairment. *Am J Hum Genet* 91: 636–645

Sebe JY, Cho S, Sheets L, Rutherford MA, von Gersdorff H & Raible DW (2017) Ca2+-Permeable AMPARs Mediate Glutamatergic Transmission and Excitotoxic Damage at the Hair Cell Ribbon Synapse. *J Neurosci* 37: 6162–6175

Sheets L, He XJ, Olt J, Schreck M, Petralia RS, Wang Y-X, Zhang Q, Beirl A, Nicolson T, Marcotti W, *et al* (2017) Enlargement of Ribbons in Zebrafish Hair Cells Increases Calcium Currents But Disrupts Afferent Spontaneous Activity and Timing of Stimulus Onset. *J Neurosci* 37: 6299–6313

Strenzke N, Chanda S, Kopp-Scheinpflug C, Khimich D, Reim K, Bulankina AV, Neef A, Wolf F, Brose N, Xu-Friedman MA, *et al* (2009) Complexin-I Is Required for High-Fidelity Transmission at the Endbulb of Held Auditory Synapse. *J Neurosci* 29: 7991–8004

Tertrais M, Bouleau Y, Emptoz A, Belleudy S, Sutton RB, Petit C, Safieddine S & Dulon D (2019) Viral Transfer of Mini-Otoferlins Partially Restores the Fast Component of Exocytosis and Uncovers Ultrafast Endocytosis in Auditory Hair Cells of Otoferlin Knock-Out Mice. *J Neurosci* 39: 3394–3411

Trapani JG & Nicolson T (2011) Mechanism of Spontaneous Activity in Afferent Neurons of the Zebrafish Lateral-Line Organ. *The Journal of Neuroscience* 31: 1614–1623

Tycko J, Wainberg M, Marinov GK, Ursu O, Hess GT, Ego BK, Aradhana, Li A, Truong A, Trevino AE, *et al* (2019) Mitigation of off-target toxicity in CRISPR-Cas9 screens for essential non-coding elements. *Nat Commun* 10: 4063

Vogl C, Cooper BH, Neef J, Wojcik SM, Reim K, Reisinger E, Brose N, Rhee J-S, Moser T & Wichmann C (2015) Unconventional molecular regulation of synaptic vesicle replenishment in cochlear inner hair cells. *J Cell Sci* 128: 638–644

Wong AB, Rutherford MA, Gabrielaitis M, Pangršič T, Göttfert F, Frank T, Michanski S, Hell S, Wolf F, Wichmann C, *et al* (2014) Developmental refinement of hair cell synapses tightens the coupling of Ca2+ influx to exocytosis. *EMBO J* 33: 247–264

Wong H-TC, Zhang Q, Beirl AJ, Petralia RS, Wang Y-X & Kindt K (2019) Synaptic mitochondria regulate hair-cell synapse size and function. *Elife* 8: e48914

Yasunaga S, Grati M, Cohen-Salmon M, El-Amraoui A, Mustapha M, Salem N, El-Zir E, Loiselet J & Petit C (1999) A mutation in OTOF, encoding otoferlin, a FER-1-like protein, causes DFNB9, a nonsyndromic form of deafness. *Nat Genet* 21: 363–369
